# Enhanced solar water oxidation and unassisted water splitting using graphite-protected bulk heterojunction organic photoactive layers

**DOI:** 10.1038/s41560-025-01736-6

**Published:** 2025-03-18

**Authors:** Matyas Daboczi, Flurin Eisner, Joel Luke, Shi Wei Yuan, Noof Al Lawati, Maoqing Zhi, Mengya Yang, Jolanda Simone Müller, Katherine Stewart, Ji-Seon Kim, Jenny Nelson, Salvador Eslava

**Affiliations:** 1 Department of Chemical Engineering and Centre for Processable Electronics, Imperial College London, London, UK; 2 Institute of Technical Physics and Materials Science, HUN-REN Centre for Energy Research, Budapest, Hungary; 3 Department of Materials, Imperial College London, London, UK; 4 School of Engineering and Materials Science, Queen Mary University of London, London, UK; 5 Department of Physics and Centre for Processable Electronics, Imperial College London, London, UK

**Keywords:** Photocatalysis, Solar fuels, Energy, Devices for energy harvesting, Chemical engineering

## Abstract

Polymer donors and non-fullerene acceptors have played an important role as photoactive materials in the development of high-efficiency organic solar cells and have immense potential in devices for direct solar hydrogen generation. However, their use in direct solar water-splitting devices has been limited by their instability in aqueous environment and recombination losses at the interface with catalysts. Here we report anodes containing PM6:D18:L8-BO photoactive layers reaching high solar water oxidation photocurrent density over 25 mA cm^−2^ at +1.23 V versus reversible hydrogen electrode and days-long operational stability. This was achieved by integrating the organic photoactive layer with a graphite sheet functionalized with earth-abundant NiFeOOH water oxidation catalyst, which provides both water resistance and electrical connection between the catalyst and the photoactive layer without any losses. Using monolithic tandem anodes containing organic PM6:D18:L8-BO and PTQ10:GS-ISO photoactive layers, we achieve a solar-to-hydrogen efficiency of 5%. These results pave the way towards high-efficiency, stable and unassisted solar hydrogen generation by low-cost organic photoactive materials.

## Abstract (web summary)

The use of organic photoactive materials in direct solar water-splitting devices has been limited by recombination losses and their instability in aqueous media. Daboczi et al. report that stable and efficient devices based on organic photoactive layers can be achieved through the application of catalyst-functionalized protective graphite sheets.

## Main

One of the most pressing challenges in achieving net zero is the production of inexpensive low-emission fuels^1^. Direct solar water splitting is a promising route towards off-grid, low-cost hydrogen production with a low environmental footprint^2^. In particular, photoelectrochemical (PEC) cells can offer sustainable, low-cost systems with high solar-to-hydrogen (STH) efficiency^3,4^. However, existing PEC systems have thus far failed to achieve the combination of high performance, long-term stability, low manufacturing cost and use of earth-abundant materials^5,6^.

Organic semiconductors are compatible with eco-friendly and low-cost large-scale manufacturing methods^7–9^ and are also promising candidates for efficiently driving PEC reactions due to their tunable optoelectronic properties^10–12^. Recently, building on high-performing donor–acceptor bulk heterojunction (BHJ) organic photovoltaic (OPV) devices, encouraging progress has been achieved on both BHJ photocathodes and photoanodes^13–18^. However, despite these demonstrations, there has been limited success in translating the high photocurrent densities (*j*_ph_) achievable in BHJ OPV devices (>25 mA cm^−2^) into PEC cells (2–15 mA cm^−2^), especially in conjunction with operational stability on the order of days^18,19^.

A major challenge to improving solar-to-fuel conversion efficiencies in organic PEC cells is minimizing electronic losses between the photo-absorbing organic semiconductor and the electrocatalyst^14,15,19^. This is particularly important for photoanodes, where the sluggish oxidation reaction kinetics can lead to rapid build-up of charges at the organic semiconductor–electrocatalyst interface that decrease efficiency and lead to degradation of the organic semiconductor^10,14^. Previous efforts to address this have included using polymer^14,16^ or metal-oxide^20,21^ interlayers, as well as more elaborate (and expensive) encapsulation techniques^17^. All these cited PEC devices apply a fully integrated structure, different to wired photovoltaic–electrolyser devices. These devices are commonly denoted as ‘photoanodes’ or ‘photoelectrodes’, despite the integration of further semiconductor and/or highly conductive layers on top of the photoactive material, which differentiates them from the traditional photoelectrodes comprising a direct semiconductor–electrolyte interface. To distinguish these traditional photoanodes from photovoltaics integrated into monolithic anodes using protection layers, we introduce the term integrated photovoltaic anode (IPV-anode) to refer to such integrated devices including the ones introduced in this Article (see further discussion in Supplementary Note 1).

Here, we present BHJ IPV-anodes that apply a straightforward and cost-effective approach using self-adhesive graphite sheet functionalized with an earth-abundant nickel–iron oxyhydroxide (NiFeOOH) electrocatalyst to simultaneously prevent degradation by the aqueous environment and eliminate electrical losses between the BHJ photoactive layer and the catalyst. The single-junction IPV-anodes reach a *j*_ph_ of over 25 mA cm^−2^ at +1.23 V versus reversible hydrogen electrode (V_RHE_) and days-long operational stability, and monolithic organic tandem IPV-anodes achieve unassisted (that is, bias-free) solar water splitting in a two-electrode setup with 5% STH efficiency.

## Fabrication of organic IPV-anodes

**Fig. 1:**
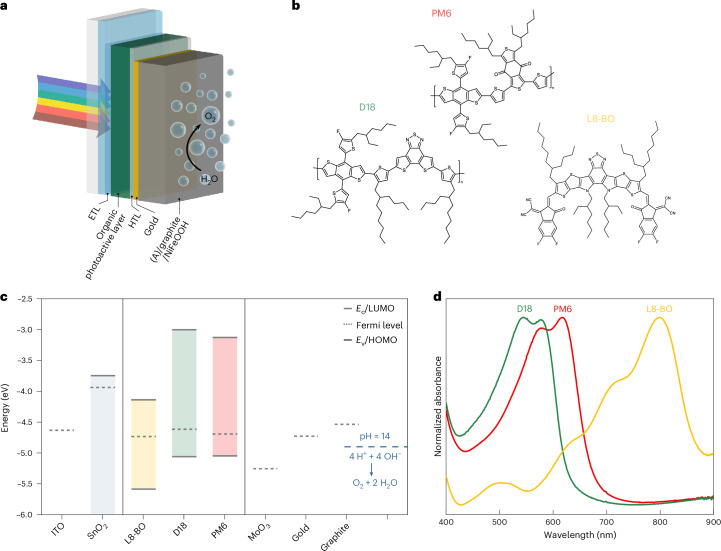
Structure, composition and energetics of the PM6:D18:L8-BO organic IPV-anodes. **a**, A schematic representation of the water oxidation IPV-anodes containing an organic photoactive layer and a self-adhesive (A) graphite sheet as a conductive protection layer (not to scale). The adhesive layer is not shown, as there is direct electrical contact between the gold and graphite layers. **b**, The chemical structures of PM6, D18 and L8-BO, the three photoactive materials in the ternary BHJ blend. **c**, The energy level diagrams for all the constituent layers of the organic IPV-anode. The shaded areas represent the bandgap of the semiconductors. The blue dashed line indicates the electrochemical potential of water oxidation to oxygen at pH 14. **d**, The normalized absorbance spectra of PM6, D18 and L8-BO. Source data

The schematic structure of the fabricated organic IPV-anodes and the related energy band diagrams and chemical composition of the materials used in optimized devices are shown in Fig. 1. In the optimized devices, a SnO_2_ electron transport layer (ETL) and a MoO_3_ hole transport layer (HTL) were used. The organic photoactive layer comprises a ternary blend of PM6 and D18 donor polymers, absorbing in the 400–700 nm wavelength range due to their relatively large optical bandgaps (1.9 and 2.1 eV, respectively), and L8-BO, a non-fullerene acceptor that absorbs photons up to 900 nm wavelength due to its narrow optical bandgap (1.45 eV). The top of the IPV-anodes consists of a 70-µm-thick graphite sheet functionalized with NiFeOOH on the front side and adhesive on the back side. This sheet is attached directly to the thin (30/10 nm) top MoO_3_/gold contact of the photoactive layer and serves simultaneously as a water-resistant and electrically conductive protective layer and electrocatalyst. The graphite sheet has highly oriented layers and large porosity (10–50 µm pores, 50% porosity), providing large surface area for the electrodeposition of the NiFeOOH electrocatalyst^22^.

The highest occupied molecular orbital (HOMO) and Fermi level values of the separate device layers were measured by ambient photoemission spectroscopy (Supplementary Fig. 1) and Kelvin probe (Supplementary Fig. 2), respectively. The energy levels of the lowest unoccupied molecular orbitals (LUMOs) for the organic semiconductors were calculated using the HOMO and the optical bandgap values to construct the energy band diagrams of all constituent layers of the IPV-anode (Fig. 1b). Importantly, the HOMO of both donor polymers (−5.05 eV) lie deeper than the theoretical electrochemical potential of water oxidation at pH 14 (−4.90 eV), a thermodynamic requirement for water oxidation and an advantage in achieving a low onset potential (*E*_on_) for photoanodes.

## Solar water oxidation performance of organic IPV-anodes

**Fig. 2:**
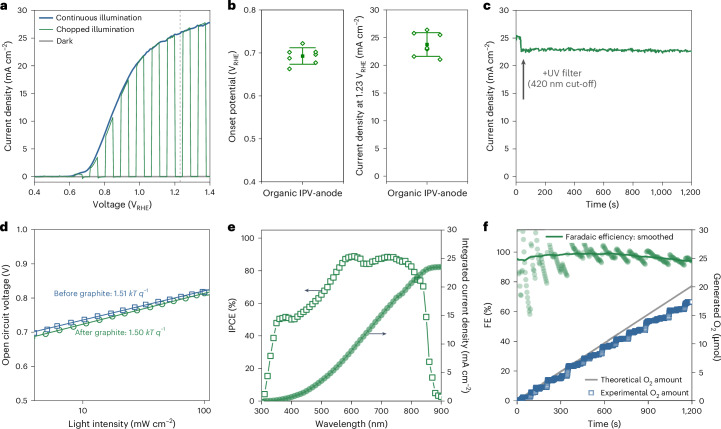
Performance of PM6:D18:L8-BO organic IPV-anodes with 0.28 cm^2^ active area measured in a PEC cell. **a**, Current density–voltage scans in the dark, under one sun continuous and chopped illumination for a high-performing PM6:D18:L8-BO IPV-anode. The scan rates were 20 mV s^−1^ for chopped illumination and 50 mV s^−1^ for the rest. The vertical dashed line at +1.23 V_RHE_ indicates the standard oxidation potential of water to oxygen. **b**, The distribution of the onset potentials and photocurrent densities at +1.23 V_RHE_ extracted from the current–voltage scans of seven organic IPV-anodes from different batches. The open diamond symbols show the values measured for the individual devices (lateral displacement is only for clarity), the solid squares are the mean values and the whiskers represent one standard deviation. **c**, The stabilized photocurrent density of a representative PM6:D18:L8-BO IPV-anode under 1 sun illumination without (0–30 s) and with (30–1,200 s) a 420 nm cut-off UV filter. **d**, The light-intensity-dependent open circuit voltage of the organic IPV-anode before and after deposition of the top graphite sheet. The open symbols represent the measured data points, while the solid lines show fitting by least squares regression. **e**, The IPCE spectra and integrated photocurrent density at +1.23 V_RHE_ of a representative organic IPV-anode. **f**, The Faradaic efficiency of an organic IPV-anode calculated from the measured amount of generated O_2_ compared with the theoretical amount of O_2_ based on the recorded photocurrent. The green solid circles represent the Faradaic efficiency values, while the green solid line shows the same data smoothed with percentile filter method using points of window of 600. The measurements were performed in an aqueous 1 M NaOH electrolyte in a PEC cell. Source data

The successful electrodeposition of the NiFeOOH electrocatalyst on the graphite sheet was confirmed by a more than 500 mV cathodic shift in *E*_on_ for water oxidation at pH 14 (Supplementary Fig. 3). These functionalized graphite sheets were attached to the thin metal electrode of OPVs, which include various HTLs and ETLs (Supplementary Figs. 4 and 5). We found that the SnO_2_ ETL and MoO_3_ HTL yielded the highest *j*_ph_ at +1.23 V_RHE_, plausibly due to the improved charge carrier extraction and higher photovoltage resulting in a reduced *E*_on_. The increased photovoltage is also reflected in the larger change in open circuit potential (ΔOCP) of the IPV-anodes upon switching off the 1 sun illumination (Supplementary Figs. 4 and 5). The optimized 0.28 cm^2^ active area IPV-anodes showed a champion *j*_ph_ of 26.4 mA cm^2^ (average of 23.8 ± 2.1 mA cm^2^) at +1.23 V_RHE_ and an *E*_on_ as low as +0.66 V_RHE_ (average of +0.69 ± 0.02 V_RHE_) both under continuous and chopped 1 sun illumination, in aqueous 1 M NaOH electrolyte at pH 14 (Fig. 2a,b). *E*_on_ was determined conservatively by linear fitting of the photocurrent rise. The saturation of *j*_ph_ above +1.4 V_RHE_ was confirmed by subtracting the dark current density from the current density under 1 sun illumination (Supplementary Fig. 6).

The IPV-anodes with the functionalized graphite sheet generated stable *j*_ph_ above 20 mA cm^2^, even when using a ultraviolet (UV) filter (Fig. 2c). This is in stark contrast with the two orders of magnitude lower *j*_ph_ (~0.2 mA cm^2^) generated by a device lacking the graphite sheet. While devices with a thicker gold layer of 100 nm achieved an initial *j*_ph_ of 9.4 mA cm^2^, they fully degraded within 15 min without the graphite sheet (Supplementary Fig. 7). These control measurements demonstrate the essential role of the graphite sheet in protecting the photoactive layer from degradation by the aqueous electrolyte. Similarly, devices with only the graphite sheet, but without MoO_3_–Au layers showed negligible *j*_ph_, highlighting the necessary role of these layers in providing effective charge extraction (Supplementary Fig. 7). The short-circuit current density (*J*_sc_) of up to 26.8 mA cm^−2^ of solar cells (Supplementary Fig. 11) agrees well with the high *j*_ph_ measured in the graphite sheet containing IPV-anodes (26.4 mA cm^2^).

Recombination losses in the organic solar cells with and without the graphite sheet were investigated by light-intensity-dependent open circuit voltage, *V*_oc_, (Fig. 2d) and *J*_sc_ (Supplementary Fig. 8) measurements. Deposition of the graphite sheet had minimal effect on the light-intensity dependence of solar cells, which suggests the elimination of most electrical losses between the top electrode of the organic BHJ solar cell and the catalyst-functionalized graphite sheet. This was confirmed by the almost identical electroluminescence, external quantum efficiency spectra (Supplementary Fig. 9), current–voltage curves (Supplementary Fig. 10) and voltage losses (Supplementary Table 1). These results show that the predeposited adhesive layer at the back of the graphite sheet is thin and/or discontinuous enough to ensure direct electrical contact between the rough graphite sheet and the Au top electrode; therefore, no conductive fillers are needed in the adhesive layer^23^. Importantly, these results demonstrate that the addition of the graphite sheet with its adhesive and NiFeOOH layers does not significantly impact charge recombination.

A necessary requirement to achieve a high *j*_ph_ is the preparation of uniform, pinhole-free photoactive layers with high shunt resistance (measured between the bottom indium tin oxide (ITO) and top graphite sheet electrodes in this case). We found that increasing the thickness of the photoactive layer from around 110 nm (the optimum for solar cells) to above 200 nm significantly enhanced performance by increasing shunt resistance (Supplementary Fig. 12). The relatively large standard deviation (±2.1 mA cm^−2^) of *j*_ph_ measured at +1.23 V_RHE_ for seven devices from different batches (Supplementary Fig. 13) is potentially due to the effect of varying active layer uniformity and resistance, especially for larger (0.28 cm^2^) devices. The statistical analysis of smaller area (0.05 cm^2^) OPV also shows a similar trend: small variation in *V*_oc_ (0.89 ± 0.01 V) but larger in *J*_sc_ (25.1 ± 0.9 mA cm^−2^) and fill factor (0.63 ± 0.03) (Supplementary Fig. 11), suggesting that the effect of active layer uniformity and shunt resistance is also present in the solar cells. Overall, the statistical analysis of IPV-anodes and solar cells confirm the high *j*_ph_ achieved close to the theoretical limit (~31 mA cm^−2^)^24^. Accordingly, the incident photon-to-current efficiency (IPCE) at +1.23 V_RHE_ of the organic IPV-anodes reached close to 90% in the 600–800 nm wavelength range, with an integrated *j*_ph_ of 23.5 mA cm^−2^ (Fig. 2e). The amount of generated O_2_ gas during continuous operation on this IPV-anode was measured, yielding a high Faradaic efficiency of 97% (Fig. 2f).

Polymer:polymer IPV-anodes were also fabricated with an organic photoactive layer comprising the polymers PM6 and PY-IT. These polymer:polymer IPV-anodes also achieved a high average *j*_ph_ of 23.3 mA cm^−2^ at +1.23 V_RHE_ and an *E*_on_ of +0.63 V_RHE_, which is a 60 mV improvement compared with the ternary PM6:D18:L8-BO devices (Supplementary Fig. 14). The lower *E*_on_ is in accordance with the higher photovoltage indicated by ΔOCP measurements (Supplementary Fig. 14), which results from reduced non-radiative voltage losses in these polymer:polymer solar cells^25^.

## Differences between PEC and solar cell operation

**Fig. 3:**
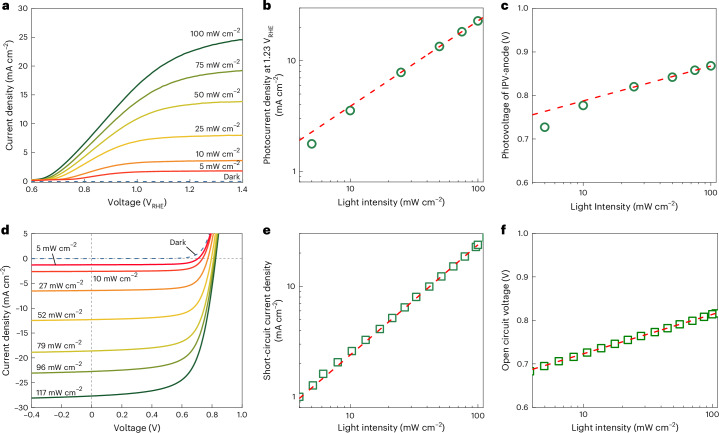
Light-intensity dependence of PM6:D18:L8-BO organic IPV-anodes and solar cells. **a**, The current density–voltage scans of the PM6:D18:L8-BO IPV-anode at different light intensities measured in aqueous 1 M NaOH electrolyte at a 50 mV s^−1^ scan rate. **b**, The photocurrent density at +1.23 V_RHE_ extracted from the current–voltage curves. **c**, The photovoltage of organic IPV-anode calculated from the onset potential shift under illumination by different light intensities compared with the dark current–voltage scan of the reference catalytic sheet. **d**, The current density–voltage scans of PM6:D18:L8-BO organic solar cells at different light intensities and a 40 mV s^−1^ scan rate. **e**,**f**, The light-intensity dependence of short-circuit current density (**e**) and open circuit voltage (**f**) of the organic solar cells. The open symbols represent the measured data points, while the solid lines show fitting by least squares regression. Source data

To gain insight into the operation and losses of the organic IPV-anodes, light-intensity-dependent measurements were performed on the devices as IPV-anodes in PEC cells and as solar cells (Fig. 3). The *j*_ph_ of organic IPV-anodes at +1.23 V_RHE_ and the *J*_sc_ of organic solar cells both show a linear relationship on a log–log scale against the light intensity. This relationship remains true for the full range of light intensities for the solar cells; however, the *j*_ph_ of the IPV-anodes fall below the power-law fit at light intensities lower than 30 mW cm^−2^ (Fig. 3b,e). This implies increased recombination in the IPV-anodes at lower photogenerated charge carrier densities, possibly originating from the electrocatalyst–electrolyte interface. A similar trend is observed in the IPV-anode photovoltage and solar cell *V*_oc_ with light intensity (Fig. 3c,f). The photovoltage of the organic IPV-anode was estimated by the onset potential shift under illumination compared with the water oxidation onset potential measured in dark for the NiFeOOH-functionalized graphite sheet. The onset potential values were extracted from the first-order derivative at 3 mA cm^−2^ V^−1^ (Supplementary Fig. 15)^26^. The light-intensity dependence of *V*_oc_ again shows a linear relationship on a semi-log scale for the organic solar cells with a slope of 1.50 *kT* *q*^−1^ (meaning a light ideality factor of 1.50), where *kT* is the thermal energy and *q* is the elementary charge. However, the relationship of the IPV-anode photovoltage and the light intensity follows the power-law fit only at high light intensities (>30 mW cm^−2^) with a slope of 1.54 *kT* *q−1*, which significantly increases at lower light intensities (up to 2.81 *kT* *q*^−1^). This reveals only slight difference under 1 sun illumination but increased recombination at low light intensities (<30 mW cm^−2^) in the organic IPV-anodes compared with the solar cells, however^27,28^.

Considering the same composition and structure of the solar cell and IPV-anode devices, the distinctly different light-intensity dependence points to the IPV-anode–electrolyte interface as the origin of increased recombination. In a solar cell applying a contact layer with deep enough work function, there is straightforward extraction of photogenerated holes even at low charge carrier densities. However, IPV-anode charge extraction (that is, injection into the electrolyte) happens only via multiple reaction steps; for example, water oxidation and oxygen evolution steps involve four holes per O_2_ molecule, which all occur in the presented IPV-anodes as confirmed by the high Faradaic efficiency (Fig. 2f). The kinetics as well as the oxidation potential of the reaction with respect to the energetics of the IPV-anode could influence the charge extraction and recombination rate at the IPV-anode–electrolyte interface, which we investigated by performing light-intensity-dependent ΔOCP measurements that allow the minimization of the kinetic influence^29^ by adding a Na_2_SO_3_ hole scavenger to the alkaline electrolyte and by reducing the pH of the electrolyte from 14 to 10 (Supplementary Figs. 16–21). The results reveal that the value of ΔOCP relates closely to the photovoltage generated by the IPV-anode and that it is not influenced by the mixed potential measured at OCP in dark^30^. More importantly, the results confirm that the charge injection at the IPV-anode–electrolyte interface is limited during oxygen evolution reaction due to insufficient energetic driving force at low light intensities (see Supplementary Note 2 for a detailed discussion). Application of deeper energy level photoactive materials (especially deeper HOMO of the donor) in organic IPV-anodes could potentially reduce such recombination at lower light intensities.

## Stability of organic IPV-anodes

**Fig. 4:**
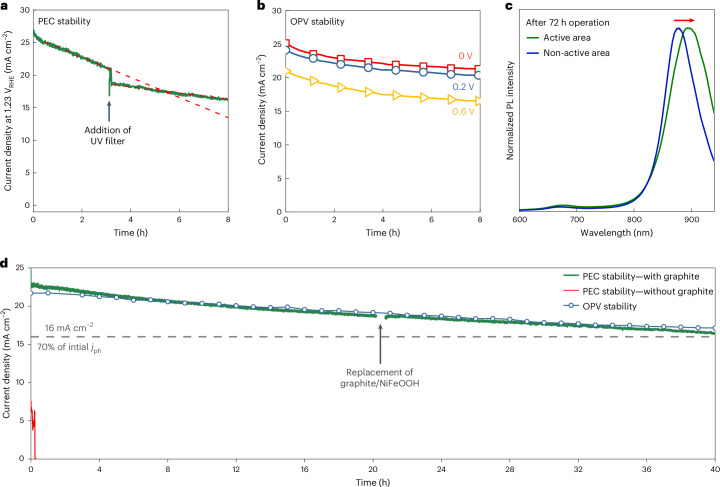
Operational stability of PM6:D18:L8-BO organic IPV-anodes in PEC cells and as solar cells at ambient conditions. **a**, A comparison of operational photocurrent stability of the organic IPV-anode at +1.23 V_RHE_ under 1 sun illumination without and with a 420 nm cut-off UV filter measured in a PEC cell. The red dashed lines show the linear fits by least squares regression to the two phases of photocurrent decay. **b**, The photocurrent stability of the organic IPV-anode measured as solar cell (OPV stability) under continuous illumination without UV filter. **c**, The normalized PL spectra of the photoactive layer of a full organic IPV-anode after 72 h of continuous operation at +1.23 V_RHE_ in a PEC cell. The red arrow indicates the shift in peak position between the PL spectra recorded within the 0.28 cm^2^ active area (illuminated under operation) and outside of the active area (not illuminated). **d**, The operational photocurrent stability of an OPV at 0.2 V and an organic IPV-anode for 40 h under 1 sun illumination at +1.23 V_RHE_ with the use of a 420 nm cut-off UV filter with and without applying graphite protection. The dashed horizontal line indicates 70% of the initial *j*_ph_ of the organic IPV-anode. After 22 h of operation, the NiFeOOH-functionalized 150-µm-thick graphite sheet was replaced with a fresh one. The PEC cell electrolyte was aqueous 1 M NaOH. Source data

The developed graphite-protected PM6:D18:L8-BO IPV-anodes were tested in lab ambient conditions and under different operational conditions to understand their degradation mechanisms and improve their stability. The high initial *j*_ph_ at +1.23 V_RHE_ applied potential was found to decay linearly after the first 0.5 h at a rate of 1.5 mA cm^−2^ h^−1^ under 1 sun illumination, which slowed down to 0.5 mA cm^−2^ h^−1^ when applying a UV filter (Fig. 4a). This detrimental UV light-driven degradation has been widely reported in OPV devices and assigned to degradation of the BHJ photoactive layer triggered by adjacent metal-oxide transport layers. This light-induced degradation is slower but still present with SnO_2_ ETL^31–33^. Measurement of the IPV-anodes as solar cells for 8 h under continuous illumination (without a UV filter) showed a roughly 15% decay of the initial photocurrent (Fig. 4b and Supplementary Fig. 22). These results suggest that a significant amount of *j*_ph_ decay originates from the photoinduced degradation of the organic photoactive layer and not from the graphite sheet interface or the presence of aqueous electrolyte.

Photoluminescence (PL) spectra recorded after 72 h of PEC operation within and outside the active area further confirm the degradation of the organic absorber layer (Fig. 4c). The active area is defined as the area illuminated (back side) and in contact with electrolyte (front side) during operation. In both areas, the PL emission is dominated by L8-BO (880 nm) due to effective energy transfer to the narrower bandgap component, although a small contribution from polymer emission can also be observed at 650–700 nm. The L8-BO emission peak is red shifted within the active area compared with outside, which suggests enhanced acceptor crystallinity and indicates phase separation between L8-BO acceptor and polymer donor. Such phase separation leads to less intermixed blend morphology, which will reduce charge generation, possibly leading to the observed loss of photocurrent. The fresh IPV-anodes before operation show no PL peak shift between the different areas of the device, confirming the PL changes observed are due to prolonged photoexcitation of the device (Supplementary Fig. 23)^34,35^.

The photodegradation of PM6 and D18 as well as polymer:polymer films was also investigated, as they are known to degrade rapidly in air under illumination (Supplementary Figs. 24–26)^36^. Based on the Raman spectra of degraded devices and UV–visible light spectra of ex situ degraded photoactive layers, we conclude that the morphological instability of these organic blend IPV-anodes is the main mechanism affecting operational stability in PEC cells (see Supplementary Note 3 for a detailed discussion).

The loss of NiFeOOH catalyst during continuous operation from the surface of the graphite sheet is evident from the accelerated photocurrent decay after 16–20 h (Supplementary Fig. 27). This is confirmed by X-ray photoelectron spectroscopy (XPS) measurements, by the recovery of photocurrent when replacing the NiFeOOH-functionalized graphite sheet and by the presence of visible graphite particles in the electrolyte (Supplementary Figs. 28–32 and Supplementary Note 4). We tested a thicker, porous graphite sheet (150 µm thick) with a thicker layer of NiFeOOH, which helped minimizing losses due to catalyst degradation (Supplementary Fig. 33). Based on these results, we also fabricated PM6:D18:L8-BO IPV-anodes with a thicker NiFeOOH catalyst layer and measured its long-term stability, as shown in Fig. 4d. These IPV-anodes showed an unprecedented 40 h long operational water oxidation stability at +1.23 V_RHE_ with *j*_ph_ maintained at or above 16 mA cm^2^ (70% of initial *j*_ph_)_._ Such long stability was achieved by application of a UV filter and replacement of the catalyst-functionalized graphite sheet after 22 h (Fig. 4d).

These results allow the identification of four phases of IPV-anode degradation: a first burn-in phase in the first hour (more pronounced with UV light) is followed by a second, mostly linear *j*_ph_ decay related solely to the morphological instability of the photoactive layer as discussed above (Fig. 4c) and as demonstrated by the nearly perfect overlap of the *j*_ph_ decay profiles of the IPV-anode and an OPV with the same structure (Fig. 4d). Third, an accelerated photocurrent decay (after 22–26 h of continuous operation) is due to the loss of most of the NiFeOOH from the surface of the graphite sheet (Supplementary Fig. 27). Fourth, if the graphite sheet is not replaced or covered with a new one, the final phase occurs, which is the non-reversible, fast degradation (reportedly due to delamination of the otherwise photochemically stable organic materials)^14^ by the aqueous electrolyte reaching the organic BHJ layer through the deteriorated graphite sheet (Supplementary Fig. 27).

The degradation of the graphite sheet was monitored by the water contact angle, which decreased from 86° to 55° over 72 h of continuous operation (Supplementary Fig. 34). In addition, scanning electron microscopy revealed significant morphological changes on the surface of the graphite, while the cross section remained unchanged (Supplementary Figs. 35 and 36). This catastrophic degradation was circumvented by repeated addition of a new graphite sheet upon signs of deterioration, which allowed demonstration of operational stability over 48 h (Supplementary Fig. 27). Alternatively, applying a combination of a denser (15% porosity) non-functionalized graphite sheet and a less dense (50% porosity) NiFeOOH-functionalized top graphite sheet allowed for 72 h long continuous water oxidation by the organic IPV-anodes and up to 100 h stability when replacing the graphite sheet twice (Supplementary Fig. 27). Recording the polarization curve of the IPV-anode before and after 72 h of solar water splitting reveals 38% reduction of *j*_ph_ at +1.23 V_RHE_ but only small change in the *E*_on_ (from +0.68 V_RHE_ to +0.62 V_RHE_), further confirming that the operation of the IPV-anode in this case is mainly limited by the morphological instability of the organic BHJ layer (Supplementary Fig. 37). IPV-anodes containing polymer:polymer photoactive layers showed stability results comparable with the ternary PM6:D18:L8-BO devices, (Supplementary Fig. 38) confirming the general applicability of the understanding gained on the degradation mechanism of these organic IPV-anodes.

## Comparison with state-of-the-art photoanodes and IPV-anodes

**Fig. 5:**
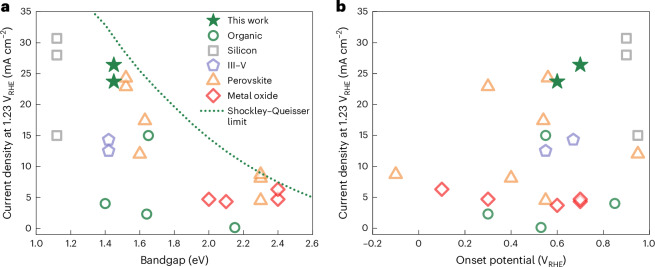
Performance comparison of a selection of reported single-junction fully integrated devices for solar water oxidation in PEC cells without any sacrificial agent. **a**,**b**, A comparison of reported photocurrent densities at +1.23 V_RHE_ as a function of the photoactive layer bandgap (**a**) and onset potential for linear rise of photocurrent (**b**). The Shockley–Queisser limit of photocurrent density with different semiconductor bandgaps is shown with a green dotted line. III–V, compound semiconductors containing elements from group III and group V of the periodic table.The performance parameters and composition of the compared devices are detailed in Supplementary Table 2 (refs. ^14,16,17,22,37,38,43–57^). Source data

Figure 5 displays a comparison of *j*_ph_ at +1.23 V_RHE_ reported for state-of-the-art fully integrated solar water oxidation devices (including both traditional photoanodes and IPV-anodes) without using any sacrificial agent. Most of the devices comprising organic semiconductors before this work generated a *j*_ph_ below 5 mA cm^−2^, apart from one report demonstrating 15 mA cm^−2^ with an organic IPV-anode^14,16,17^. The highest *j*_ph_ of the PM6:D18:L8-BO and PM6:PY-IT devices presented in this Article (26.4 mA cm^−2^ and 23.7 mA cm^−2^, respectively) mean a significant leap forward, showcasing the first organic IPV-anode with a *j*_ph_ above 25 mA cm^−2^. Importantly, these high *j*_ph_ values are accompanied by days-long operational water oxidation stability in PEC cells, which is also a large improvement compared with previous organic photoanodes and IPV-anodes reporting stability of minutes or hours (Supplementary Table 2). Even when compared with IPV-anodes with different photoactive layers such as perovskites or silicon, the PM6:D18:L8-BO organic IPV-anode offers one of the highest *j*_ph_ so far for its given bandgap (Fig. 5a). The only reported IPV-anodes generating higher a *j*_ph_ are based on silicon photoactive layers, but these show a high *E*_on_ of +0.9 V_RHE_ or above due to their smaller 1.1 eV bandgap (Fig. 5b)^37,38^.

## Tandem organic IPV-anodes for unassisted operation

**Fig. 6:**
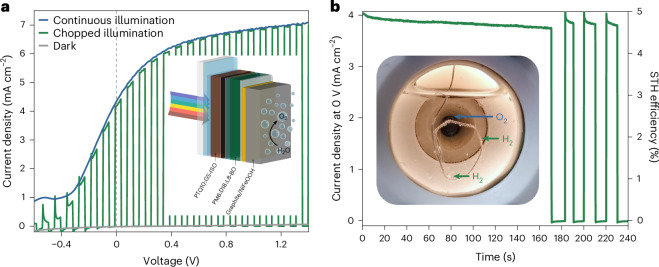
Performance of the monolithic organic tandem IPV-anode with PM6:D18:L8-BO and PTQ10:GS-ISO absorber layers in a two-electrode PEC cell. **a**, The current density–voltage scans at a 50 mV s^−1^ scan rate in dark, under 1 sun continuous and chopped illumination for the monolithic tandem IPV-anode in a two-electrode cell with a Pt counter electrode. The scan rate for the chopped illumination was 20 mV s^−1^. The vertical dashed line indicates a zero applied potential. Inset: schematic structure of the monolithic organic tandem IPV-anode (not to scale). **b**, The unassisted, two-electrode operational PEC stability of the monolithic organic tandem under continuous (0–170 s) and chopped (170–240 s) 1 sun illumination. Inset: photograph of the tandem IPV-anode and counter electrode in unassisted operation generating O_2_ and H_2_ bubbles, respectively. The measurements were performed in a PEC cell with aqueous 1 M NaOH electrolyte. Source data

Finally, building on the developed single-junction PM6:D18:L8-BO devices, monolithic tandem IPV-anodes comprising two photoactive layers were fabricated and tested for unassisted (that is, bias-free) solar water splitting in conjunction with a platinum counter electrode (Fig. 6). The tandem cells comprise a wide-bandgap BHJ photoactive layer of GS-ISO, a non-fused non-fullerene acceptor, and PTQ10, a low synthetic complexity polymer donor with PM6:D18:L8-BO as the narrow-bandgap photo-absorber. The tandem IPV-anode shows a remarkably low *E*_on_ of −0.41 V_RHE_ in a three-electrode setup owing to its large photovoltage (Supplementary Fig. 39), which is also reflected in large ΔOCP value of 1.75 V (Supplementary Fig. 40). This allows for unassisted solar water splitting: the tandem IPV-anodes in a two-electrode setup demonstrated *j*_ph_ of 4.3 mA cm^−2^ at zero applied bias (Fig. 6a), which translates into 5% STH efficiency. The unassisted water splitting in the PEC cells was confirmed by the formation of O_2_ bubbles on the tandem organic IPV-anode and H_2_ bubbles on the counter electrode (Fig. 6b and Supplementary Video 1). The average Faradaic efficiency of the tandem IPV-anode in two-electrode, unassisted operation was 95% (Supplementary Fig. 41). Continuous operation at zero applied bias in two-electrode setup demonstrates the potential of the organic IPV-anodes for unassisted solar hydrogen generation (Fig. 6b). The application of a UV filter allowed 4 h unassisted solar water-splitting stability by the tandem device while maintaining 70% of the initial *j*_ph_ (Supplementary Fig. 42).

Comparing the tandem IPV-anode stabilities at applied biases of 0 V and tandem OPV stabilities at equivalent voltages of +1.8 V reveals that the *j*_ph_ in the IPV-anode is less stable under bias-free conditions partly due to the *j*_ph_ decay of the OPV. Accordingly, the tandem IPV-anodes demonstrate stable water oxidation at a high applied bias of +1.23 V_RHE_ in agreement with stable *j*_ph_ generated by the tandem OPV at 0 V (Supplementary Figs. 42 and 43). Interestingly, this degradation of the tandem IPV-anodes recovers after leaving them at OCP for a few hours (Supplementary Fig. 42). This recoverable degradation is partly attributed to the reversible morphological changes in the photoactive layers and is also probably influenced by the detachment of surface oxygen bubbles during the recovery period. Bubbles can alter the local pH and increase the overpotential for water oxidation, particularly when the IPV-anode is covered by large bubbles as shown in Fig. 6b and Supplementary Fig. 44 (refs. ^39,40^). More morphologically stable organic photoactive layers and management of bubbles will allow even longer unassisted solar water splitting by organic tandem IPV-anodes.

## Conclusion

The presented fully integrated organic anodes with a BHJ PM6:D18:L8-BO photoactive layer demonstrate photocurrent densities above 25 mA cm^2^ at +1.23 V_RHE_ and 40 h long operational solar water oxidation stability in PEC cells while maintaining 70% of the initial *j*_ph_. Such high performance was achieved by the application of an inexpensive graphite sheet functionalized by the earth-abundant electrocatalyst NiFeOOH that served as a conductive, water-resistant top contact without introducing any electrical losses. This approach also allowed the fabrication of anodes integrating PM6:PY-IT polymer:polymer organic photoactive layer reaching photocurrent densities above 23 mA cm^2^ at +1.23 V_RHE_ and a reduced onset potential of +0.63 V_RHE_. Furthermore, monolithic tandem anodes integrating organic PM6:D18:L8-BO and PTQ10:GS-ISO photoactive layers were prepared reaching 5% STH efficiency in a two-electrode PEC setup, which highlights the potential of integrating organic BHJ photoactive layers for stable, unassisted solar water splitting. A comparison of PEC and photovoltaic operations in terms of photocurrent stability and light-intensity-dependent performance, as well as PL measurements of the organic photoactive layer, suggest strategies for further improvement of the organic devices: the photostability of the active layer is a key factor to increase the overall operational stability and maintaining large quasi-Fermi level splitting (that is, illumination above 0.5 sun light intensity) in PEC operation is critical to avoid large recombination losses, while management of bubbles will allow more stable unassisted solar water splitting.

## Methods

### PM6:D18:L8-BO and PM6:PY-IT organic device fabrication

The ITO-coated glass substrates were cleaned sequentially in sonication baths of soapy water, water, acetone and isopropanol. Tin oxide (SnO_2_) nanoparticle ink (Avantama N-31) was spin coated at 3,000 rpm onto the cleaned substrates and annealed at 200 °C for 30 min inside a glovebox. Poly((4,8-bis(5-(2-ethylhexyl)-4-fluoro-2-thienyl)benzo[1,2-*b*:4,5-*b*′]dithiophene-2,6-diyl)-2,5-thiophenediyl (5,7-bis(2-ethylhexyl)-4,8-dioxo-4*H*,8*H*-benzo[1,2-*c*:4,5-*c*′]dithiophene-1,3-diyl)-2,5-thiophenediyl)) (PM6), poly (dithieno[3,2-*e*:2′,3′-*g*]-2,1,-3-benzothiadiazole-5,8-diyl(4-(2-butyloctyl)-2,5-thiophenediyl)(4,8-bis(5-(2-ethylhexyl)-4-fluoro-2-thienyl)benzo[1,2-*b*:4,5-*b*′]dithiophene-2, *6*-diyl) (3-(2-butyloctyl)-2,5-thiophenediyl)) (D18) and 2,2′-((2*Z*,2′*Z*)-((3,9-bis(2-butyloctyl)-12,13-bis(2-ethylhexyl)-12,13-dihydro-[1,2,5]thiadiazolo[3,4-*e*]thieno[2″,3″:4′,5′]thieno[2′,3′:4,5]pyrrolo[3,2-*g*]thieno[2′,3′:4,5] thieno[3,2-*b*]indole-2,10-diyl)bis(methaneylylidene))bis(5,6-difluoro-3-oxo-2,3-dihydro-1*H*-indene-2,1-diylidene))dimalononitrile (L8-BO), all from 1-Material, were dissolved at 14 mg ml^−1^ for thin devices or 20 mg ml^−1^ for thick devices in chloroform at weight ratios of 0.8:0.2:1.2, respectively, with 50% by total weight added diodobenzene and stirred for 2 h at 50 °C. The precursor solution was then spin coated at 3,000 rpm and annealed at 85 °C for 10 min on a hot plate to form the photoactive layers inside a glovebox. For PM6:PY-IT devices, PM6 and poly[(2,2′-((2*Z*,2′*Z*)-((12,13-bis(2-octyldodecyl)-3,9-diundecyl-12,13-dihydro[1,2,5]thiadiazolo[3,4-*e*]thieno[2′′,3′′:4′,5′]thieno[2′,3′:4,5] pyrrolo[3,2-*g*]thieno[2′,3′:4,5]thieno[3,2-*b*]-indole-2,10-diyl)bis(methanylylidene))bis(5-methyl-3-oxo -2,3-dihydro-1*H*-indene-2,1-diylidene))dimalononitrile-co-2,5-thiophene (PY-IT) from Solarmer were dissolved at a weight ratio of 1:1.2, respectively, at 18 mg ml^−1^ in chloroform, with 1% added chloronaphtalene, spin coated at 1,700 rpm for 30 s and annealed at 100 °C for 10 min. After PM6:D18:L8-BO or PM6:PY-IT deposition, subsequently 10 nm of MoO_3_ and 30 nm of gold (for IPV-anodes) or 100 nm of silver (for solar cells) were evaporated on top of the active layers.

### PTQ10:GS-ISO and PM6:D18:L8-BO tandem organic device fabrication

Poly[[6,7-difluoro[(2-hexyldecyl)oxy]-5,8-quinoxalinediyl]-2,5-thiophenediyl]]) (PTQ10) and GS-ISO^41^ from 1-Material were dissolved overnight at 20 mg ml^−1^ in chloroform at a weight ratio of 1:1.5, respectively. The solution was spin cast at 3,000 rpm for 30 s onto ITO-coated glass/SnO_2_ substrates and annealed at 145 °C for 25 min. Next, the samples were preheated at 120 °C, and BM-HTL-1 (Brilliant Matters), previously sonicated and filtered, was spin cast at 3,000 rpm for 50 s, followed by drying for 5 min at 100 °C. Subsequently, 1 nm of gold was evaporated; SnO_2_ N-31 ink was spin cast at 3,000 rpm for 30 s and annealed at 145 °C for 10 min in a glovebox; and D18:PM6:L8-BO, MoO_3_ and gold were deposited sequentially as described above.

### Organic IPV-anode fabrication

The devices were protected for IPV-anode use by manually attaching on top of the thin metal electrode of the OPV one or more graphite sheet(s) with an acrylic adhesive layer on the back side and NiFeOOH on the top side. An 0.070-mm graphite sheet (self-adhesive, RS, Panasonic, 1,000 W m^−1^ K^−1^, 115 mm × 90 mm) was applied and in some cases, a combination of a 0.025-mm-thick (self-adhesive, 1,600 W m^−1^ K^−1^, 115 mm × 90 mm) and 0.150-mm-thick graphite sheets was used, as stated in figure captions (Fig. 4d and Supplementary Figs. 27, 33 and 42). The NiFeOOH was previously electrodeposited on the graphite sheet(s) using an aqueous solution of 40 mM of nickel sulfate hexahydrate (Sigma-Aldrich, ≥98%) and 10 mM of iron sulfate heptahydrate (Sigma-Aldrich, ≥99%), purged with N_2_ for 30 min. A three-electrode setup with platinum counter electrode and a Ag/AgCl reference electrode was used for the electrodeposition. The potential was swept from +0.6 to +1.0 *V*_Ag/AgCl_ at 20 mV s^−1^ scan rate until the electrodeposition charge density reached 4.2 mC cm^−2^. For the control samples without the graphite sheet, NiFeOOH was electrodeposited on top of a 30- or 100-nm-thick Au layer directly, using the PEC cell. In these cases, the electrolyte was changed in the cell, and the PEC characterization of the samples was performed immediately after electrodeposition. For the IPV-anodes with a Pt catalyst, the graphite sheets were functionalized by electrodeposition at constant −0.5 V in a three-electrode setup using the Ag/AgCl reference and Pt counter electrode, until the electrodeposition charge density reached 100 mC cm^−2^.

### Optoelectronic characterization

A Shimadzu UV-2600 spectrophotometer was used to obtain the UV–visible light spectra of the thin organic layers deposited on ITO-coated glass. Electroluminescence spectra were obtained using a 2450 Keithley source meter and a Shamrock 303 spectrograph and iDUS InGaAs array detector (Andor SR 303i-B, cooled to −90 °C). The bandgaps of individual materials and blends were calculated using the intersection between absorption and emission spectra.

The PL and Raman spectroscopy measurements on fresh and degraded devices were carried out at different sample positions using a Renishaw in Via Raman microscope with a 50× objective in backscattering configuration. A holographic notch filter was used to remove Rayleigh scattered light. A diffraction grating of 2400 l mm^−1^ was used for Raman measurements, and a grating of 300 l mm^−1^ was used for PL measurements. The laser spot (*λ* = 514 nm, *d* ≈ 10 µm) was focused on to the photoactive layer through the glass substrate (through the back side of the sample with respect to the top, non-transparent graphite sheet side), and the power was adjusted to give an adequate signal while avoiding laser induced degradation; laser power was maintained between samples to allow comparison. During measurement, the samples were held in a Linkam stage under a flow of nitrogen to inhibit photooxidation.

### (Photo)electrochemical and solar cell measurements

The (photo)electrodes were characterized in a three-electrode setup using an Ivium Compacstat potentiostat, platinum counter electrode, KCl-saturated Ag/AgCl reference electrode and an aqueous 1 M sodium hydroxide electrolyte, sometimes with the addition of Na_2_SO_3_ (0.2 M) and H_2_O_2_ (0.5 M). Lot Quantum Design xenon lamp was applied as illumination source with an AM 1.5 G filter and a circular mask with an area of 0.28 cm^2^. A 1 sun (100 mW cm^−2^) irradiance was calibrated by a certified International Light Technologies SEL623 photodetector. The equation *V*_RHE_ = *V*_Ag/AgCl_ + 0.0592 × pH + 0.1976 was used to convert the potentials applied versus the Ag/AgCl reference electrode (*V*_Ag/AgCl_) to applied potentials versus the reversible hydrogen electrode (*V*_RHE_). The potential of the reference electrode (*V*_Ag/AgCl_) was 0.1976 V versus the standard hydrogen electrode. The OCP of the photoelectrodes were measured under 1 sun illumination for 30 s, then in dark for another 30 s, and the difference when switching the light off was calculated to provide the value of ΔOCP. The current–voltage scans of the graphite sheets attached to glass substrates with and without the NiFeOOH catalyst were recorded with the same setup without illumination. Two-electrode (photo)electrochemical measurements of the tandem devices were performed with the same setup but with only a counter (platinum) and working electrode connection unless stated differently. Both the PEC and OPV devices were scanned from forward to reverse bias (reverse scan). The photoelectrodes were also characterized as solar cells using a Keithley 2400 source voltmeter and Newport Oriel solar simulator. The solar cell degradation was studied at ambient conditions with a 10 mV s^−1^ scan rate.

The operational PEC photocurrent stability of IPV-anodes was measured under continuous 1 sun illumination, at an applied bias of +1.23 V_RHE_ for three-electrode measurements. Pyroscience FireStingO2 fibre-optic oxygen meter with a TROXROB10 oxygen probe and a TDIP temperature sensor was used to measure the percentage of oxygen (*P*_O2_) in the defined overhead volume (*V*). After correcting for the dark baseline, the measured amount of oxygen (*n*_O2,m_) generated by the IPV-anode under 1 sun illumination was calculated (*n*_O2_ = *n* × *P*_O2_) based on the measured pressure (*p*) and temperature (*T*) using the ideal gas law. A gas-tight cell was used, which was purged with nitrogen before the measurement. The amount of dissolved oxygen in the electrolyte was calculated applying Henry’s law and added to the amount measured in the overhead space. The theoretical amount of oxygen (*n*_O2,t_) was calculated for every second of the measurement from the measured photocurrent based on the equation *Q* = *n*_e−_ × *F*, where *Q* is the number of charges (C), *n*_e−_ is the number of electrons (mol) and *F* is the Faraday constant (96,485.3 C mol^−1^). The generation of 1 mol oxygen was considered to involve 4 mol of electrons. From the measured amount of oxygen and the theoretical amount of oxygen based on the measured photocurrent, the Faradaic efficiency (FE) was calculated (FE = *n*_O2,m_/*n*_O2,t_) for every second of the measurement. The onset of the measured *P*_O2_ was shifted in time to overlap with the measured photocurrent. The final value of the FE was calculated by averaging the FE values for the full plotted time of the measurement (1,200 s for the single-junction and 3,600 s for the tandem IPV-anodes). MSH-300F LOT Quantum Design monochromator was used to record the photocurrents generated under selected wavelength illumination and calculate IPCE. The intensity of the monochromatic light was measured using a certified International Light Technologies SEL033/U photodetector, which allowed us to obtain IPCE spectra in the 300–1,000 nm range. The STH efficiency of the tandem IPV-anode was calculated from the photocurrent density measured in the two-electrode setup, under 1 sun illumination without any applied bias (*j*_sc_) using the following formula: STH = |*j*_sc_| × (1.23 V) × FE/100 mW cm^−2^.

### Energy level measurements

The cube root photoemission of the samples was measured by ambient photoemission spectroscopy (APS, KP Technology, SKP5050) and extrapolated to zero, to determine the valence band edge, *E*_v_, values of the thin layers. Monochromatic UV light irradiation was scanned in the 4.4–6.4 eV range. Kelvin probe measurements were used to record contact potential difference between the tip and the sample. A cleaned silver reference was used to calibrate the tip work function, which then allowed us to determine the Fermi level of the samples taking into account the measured contact potential difference of the samples and the work function of the tip. The *E*_v_ and conduction band edge, *E*_c_, values of SnO_2_ were not measured; instead, the *E*_c_ was estimated 0.2 eV above the measured *E*_F_, which is typical for strong n-type materials^32,42^.

### Characterization of degraded samples

A Thermo Fisher K-Alpha+ X-ray photoelectron spectrometer with monochromated, micro-focused Al K_α_ X-ray source was used to obtain the XPS spectra of the fresh and degraded catalytic graphite sheets. All binding energies of all spectra were corrected using the 284.8 eV adventitious carbon peak as reference. The water contact angle of the graphite sheets was measured by a Kruss drop shape analyser (DSA25B). The contact angle was determined using the Young–Laplace method.

### Reporting summary

Further information on research design is available in the Nature Portfolio Reporting Summary linked to this article.

## Supplementary information

Supplementary Information Supplementary Notes 1–4, Figs. 1–44 and Tables 1 and 2.

Reporting Summary

Supplementary Video 1 Unassisted solar water splitting in a PEC cell by a tandem organic IPV-anode confirmed by the formation of O_2_ bubbles on the anode and H_2_ bubbles on the counter electrode.

## Source data

Source Data Fig. 1 Raw data for Fig. 1.

Source Data Fig. 2 Raw data for Fig. 2.

Source Data Fig. 3 Raw data for Fig. 3.

Source Data Fig. 4 Raw data for Fig. 4.

Source Data Fig. 5 Raw data for Fig. 5.

Source Data Fig. 6 Raw data for Fig. 6.

## Data Availability

All data generated or analysed during this study are included in the Article and its Supplementary Information. Source data are provided with this paper. These data are also available via Figshare at 10.6084/m9.figshare.28169375 (ref. ^58^).
