# Supplementary material for: Enhanced solar water oxidation and unassisted water splitting using graphite-protected bulk heterojunction organic photoactive layers
Source: Nat Energy. 2025 Mar 18;10(5):581–91. doi: 10.1038/s41560-025-01736-6 (PMC12116381; doi:10.1038/s41560-025-01736-6)
Supplement: Supplementary file 1 — Supplementary Notes 1–4, Figs. 1–44 and Tables 1 and 2. [file 41560_2025_1736_MOESM1_ESM.pdf]

nature energy

Supplementary information https://doi.org/10.1038/s41560-025-01736-6

Enhanced solar water oxidation and unassisted water splitting using graphite-protected bulk heterojunction organic photoactive layers

In the format provided by the authors and unedited

Table of Contents

1. Supplementary Note 1 2

2. Supplementary Note 2 3

3. Supplementary Note 3 4

4. Supplementary Note 4 5

5. Energy level measurements 6

6. Electrocatalytic activity of graphite sheet functionalized by NiFeOOH 7

7. Characterization of IPV-anodes with different transport and photoactive layers 8

8. Analysis of organic IPV-anode performance 9

9. Analysis of recombination of organic solar cells with and without graphite sheet 10

10. Voltage-loss analysis of organic solar cells 11

11. Analysis of solar cell performance and its distribution 12

12. Analysis of organic IPV-anode performance and its distribution 13

13. Polymer:polymer IPV-anodes 14

14. Measurements of IPV-anodes in PEC cells with and without hole scavengers 15

15. Stability of organic solar cells and photoelectrodes at ambient environment 18

16. Tandem organic IPV-anodes and solar cells 28

17. Reported solar water oxidation performance comparison of monolithic, fully integrated devices 31

18. Supplementary references 33

1. Supplementary Note 1

Devices with electrodes (anodes or cathodes) that integrate photovoltaics into a monolithic structure offer several advantages, including potential cost reductions, improved thermal management for enhanced system efficiency, and the beneficial application of photothermal effects on catalytic activity, as highlighted in previous studies.[3,46,58,59] These integrated devices differ from traditional photovoltaic-electrolyzer (PV-EC) systems, where PV modules are wired to separate electrolyzers via converters and power electronics, making thermal management more challenging. They also differ from traditional photoelectrochemical (PEC) cells, which use photoanodes and/or photocathodes with direct semiconductor/electrolyte interface. Instead, these integrated devices are in the middle of the spectrum between the two extremes of PV-EC and PEC systems, leveraging advantages from both approaches. The taxonomy of solar-driven chemical devices has been a longstanding challenge and addressing it in detail lies beyond the scope of this manuscript. Dedicated articles on this topic have been published, although no universally accepted classification has been established.[60-64] In this work, to differentiate traditional photoanodes with direct semiconductor/electrolyte interface, as well as PV-EC devices, from monolithic anodes integrating photovoltaics and electrocatalytic layers, we introduce the term “integrated photovoltaic-anode” (IPV-anode).

2. Supplementary Note 2

ΔOCP measurements are performed with zero net current at the IPV-anode/electrolyte interface and, therefore, they allow for the minimization of the kinetic influence as reported previously.[29] The light intensity dependence of ΔOCP without any sacrificial agent confirms the increased recombination at lower light intensities (<30 mW cm^{-2}) and suggests that the injection of photogenerated holes into the electrolyte is constrained by insufficient energetic driving force (Supplementary Fig. 16). The HOMO of the polymer donors lies only 150 mV deeper compared to the water oxidation potential (at pH 14 as used here). This means that at low illumination intensities the quasi-Fermi level of photogenerated holes will be shallower than the oxidation potential of water, providing insufficient driving force for the chemical reaction, leading to charge accumulation and to the observed increase in recombination.

The addition of Na_2SO_3 hole scavenger shifts the E_{on} of the organic IPV-anodes from +0.75 to +0.44 V_{RHE}. This shift agrees well with the lower E_{on} for dark catalytic activity and reflects the significantly shallower oxidation potential of Na_2SO_3 compared with the oxidation potential of OER (Supplementary Fig. 17). Importantly, the ΔOCP values were measured to be exactly the same without and with hole scavenger, which confirms that the value of ΔOCP relates closely to the photovoltage generated by the IPV-anode, and it is not influenced by the mixed potential measured at OCP in dark (Supplementary Fig. 18).[30] Light intensity dependent measurements with Na_2SO_3 show that the power-law fit of both the IPV-anode photovoltage (calculated from the first-order derivative of j_{ph} at 3 mA cm^{-2} V^{-1}) and ΔOCP is recovered even at low light intensities (Supplementary Fig. 19 and Supplementary Fig. 20). This can be rationalized by the more than 200 mV shallower oxidation potential of Na_2SO_3 compared to OER, providing sufficient energetic driving force for the oxidation reaction even at low light intensities. Therefore, the recovery of the power-law fit in the presence of Na_2SO_3 further confirms that charge injection at the IPV-anode/electrolyte interface is constrained during OER due to insufficient energetic driving force. This is also consistent with the further hindrance of charge injection at pH 10 compared with pH 14, due to the deeper water oxidation potential at pH 10, which results in a significantly reduced j_{ph} (Supplementary Fig. 21). Application of deeper energy level photoactive materials (especially deeper HOMO of the donor) in organic IPV-anodes could potentially reduce such recombination at lower light intensities.

3. Supplementary Note 3

The photodegradation of PM6 and D18 was also investigated, as they are known to degrade rapidly in air under illumination.[36] Absorption spectra of ternary blend films illuminated in air for 48 h reveal quenching of the polymeric absorption, demonstrating sensitivity of the ternary blend to this degradation mechanism (Supplementary Fig. 24). However, Raman measurements on the devices show no clear signatures of polymer photodegradation (Supplementary Fig. 25) and FT-IR measurements in previous studies have also ruled out the oxidation of the polymer backbone.[36] We rationalize this by considering that in complete devices, the active layer is partially protected from air by the top layers including the graphite sheet, slowing down this mechanism. To replicate this, photoactive layers were illuminated in nitrogen for 187 h and only small signatures of degradation in the absorption spectra were observed (Supplementary Fig. 24). Similar changes were observed for the polymer:polymer films degraded under the same conditions (Supplementary Fig. 26). Therefore, although devices undergo slow photoinduced polymer degradation, the morphological instability of these organic blend IPV-anodes is considered as the main mechanism affecting operational stability in PEC cells.

4. Supplementary Note 4

The loss of surface NiFeOOH catalyst layer is evidenced by the disappearance of the Ni 2p and Fe 2p XPS peaks after 20 h of dark operation of the catalytic sheets (Supplementary Fig. 28) and in full IPV-anodes after days of operation in a PEC cell (Supplementary Fig. 29, Supplementary Fig. 30). The photocurrent loss due to the catalyst degradation is further corroborated by the j_{ph} recovering (from 14.5 to 18 mA cm^{-2}) after the addition of a graphite sheet with freshly electrodeposited NiFeOOH (Supplementary Fig. 27). The presence of visible graphite particles in the electrolyte (Supplementary Fig. 31) and new peaks related to oxidized carbon in the C 1s and O 1s XPS spectra of the fully degraded graphite sheets (Supplementary Fig. 32) further suggest that both physical and chemical degradation of the graphite during the oxygen evolution play an important role in the decreasing catalytic activity following the loss of NiFeOOH catalysts from the graphite surface.

5. Energy level measurements

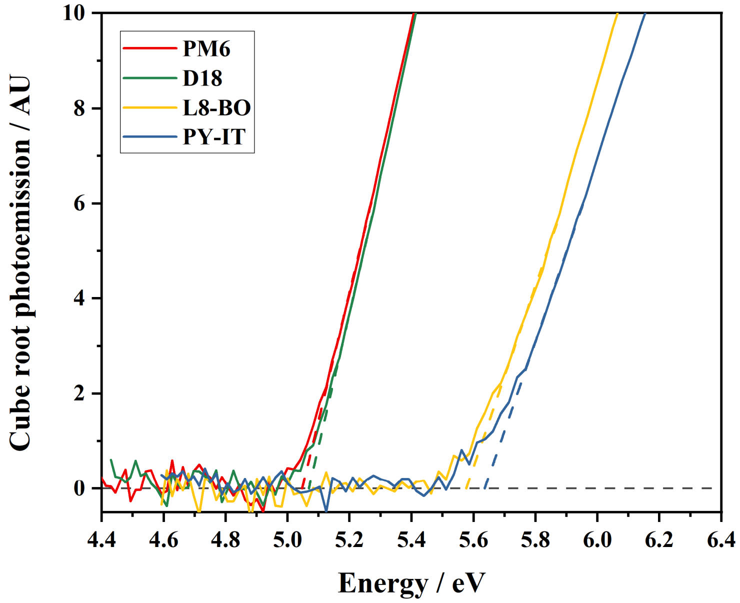

Supplementary Fig. 1 | Ambient photoemission spectra of the organic thin films. The constituents of the ternary blend were deposited separately on ITO-coated glass substrates. Dashed lines show the linear fits and extrapolation of the cube root photoemission signal, which was used to determine the highest occupied molecular orbital energy levels.

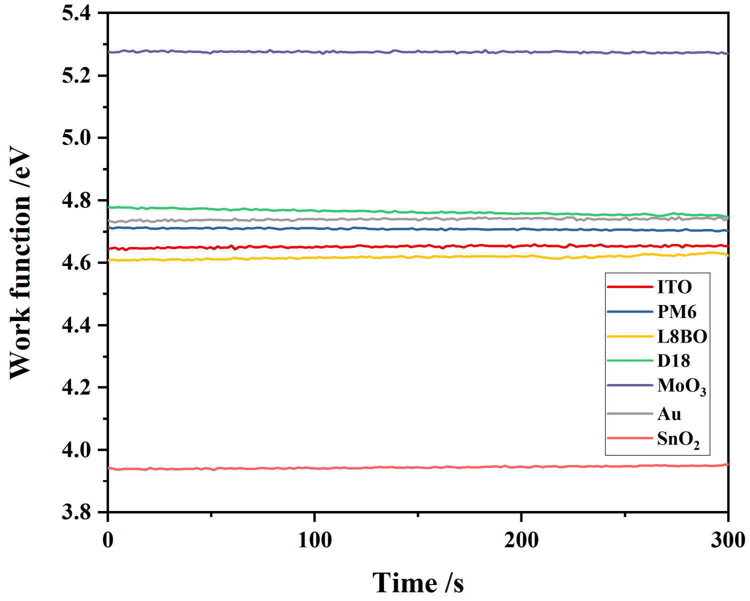

Supplementary Fig. 2 | Stable work function measurements of the constituent layers of organic IPV-anodes. The thin layers were deposited on ITO-coated glass substrates and the work function was measured by a Kelvin probe. The dark equilibrium work function value was used to calculate the Fermi level of the layers.

6. Electrocatalytic activity of graphite sheet functionalized by NiFeOOH

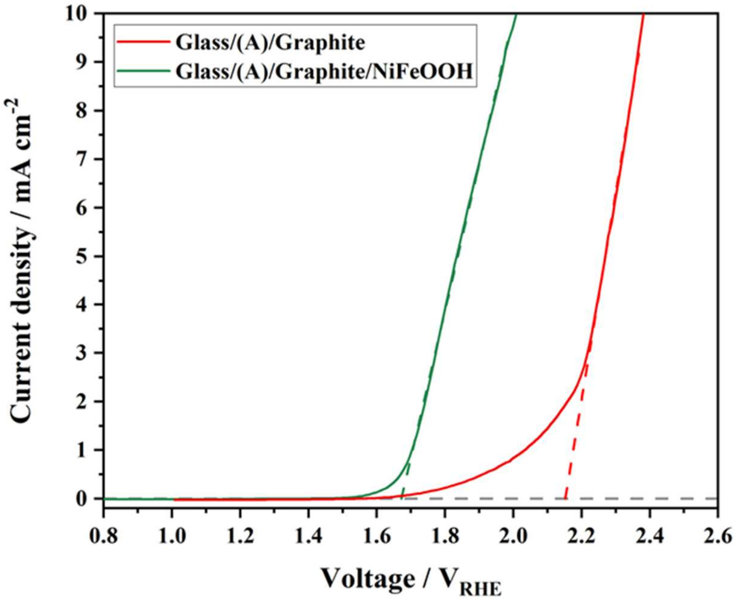

Supplementary Fig. 3 | Current–voltage scans of graphite sheets without and with electrodeposited NiFeOOH electrocatalyst. The 70 µm thick graphite sheets were attached to a glass slide to have only the top surface available for water oxidation, as in the IPV-anodes. The onset potential of water oxidation is estimated by linear fits of the current rise (dashed lines). The measurements were performed at pH 14 in an aqueous 1 M NaOH electrolyte at 50 mV s^{-1} scan rate. The measured current-voltage scans are shown without any i*R* correction.

7. Characterization of IPV-anodes with different transport and photoactive layers

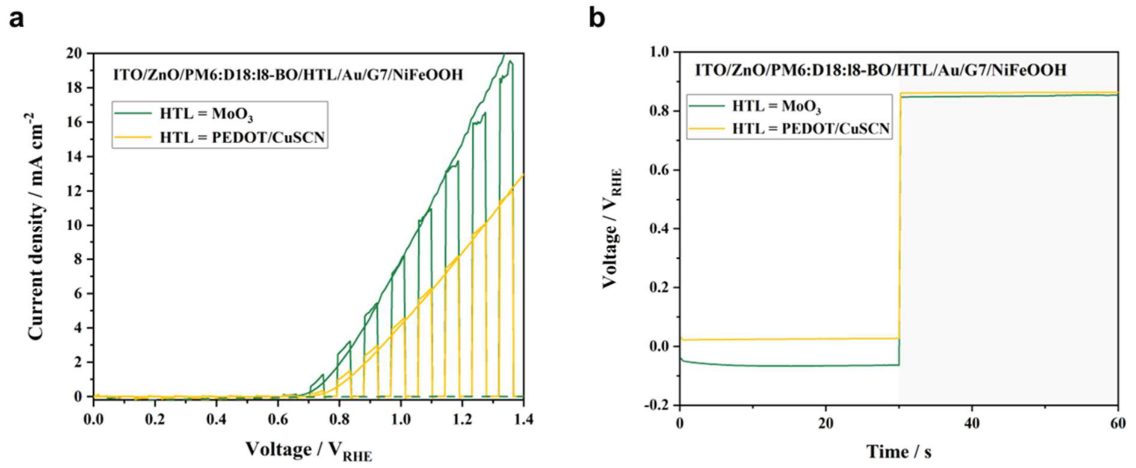

Supplementary Fig. 4 | Characterization of PM6:D18:L8-BO organic IPV-anodes in a PEC cell applying different hole transport layers (HTLs) and a thinner (110 nm) BHJ layer. a, Current–voltage scans at 50 mV s^{-1} scan rate under continuous and chopped 1 sun illumination (solid line) and in dark (dashed line). The scan rate for the chopped illumination was 20 mV s^{-1}. **b,** Open circuit potential measurements under 1 sun illumination (0 – 30 s) and in dark (30 – 60 s) of the same devices. The photoactive layers were deposited on ZnO electron transport layer in all cases. The measurements were performed in aqueous 1 M NaOH electrolyte.

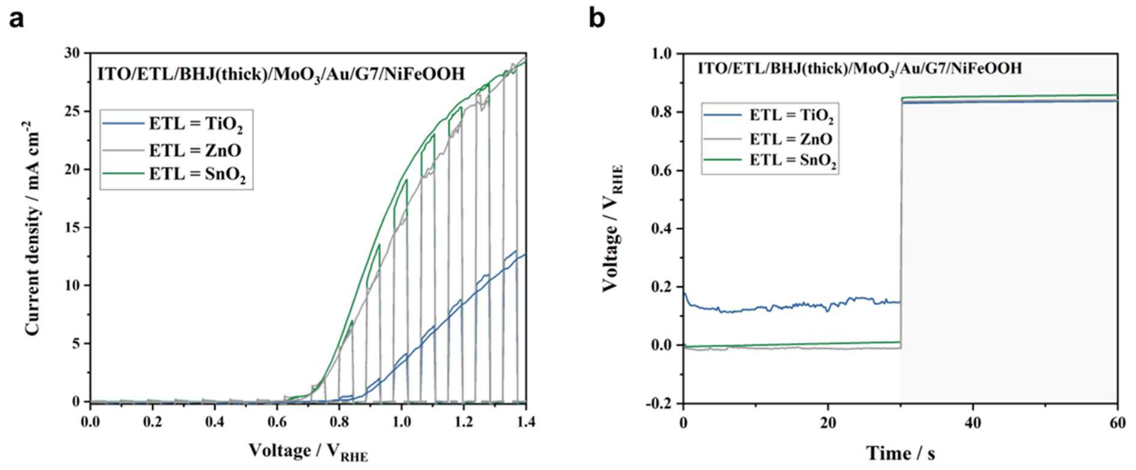

Supplementary Fig. 5 | Characterization of PM6:D18:L8-BO organic IPV-anodes in a PEC cell, applying different electron transport layers (ETLs) and a thicker (200 nm) BHJ layer. a, Current–voltage scans at 50 mV s^{-1} scan rate under continuous and chopped 1 sun illumination (solid line) and in dark (dashed line). The scan rate for the chopped illumination was 20 mV s^{-1}. **b,** Open circuit potential measurements under 1 sun illumination (0 – 30 s) and in dark (30 – 60 s) of the same devices. MoO_3 was evaporated on the photoactive layers as hole transport layer in all cases. The measurements were performed in aqueous 1 M NaOH electrolyte.

8. Analysis of organic IPV-anode performance

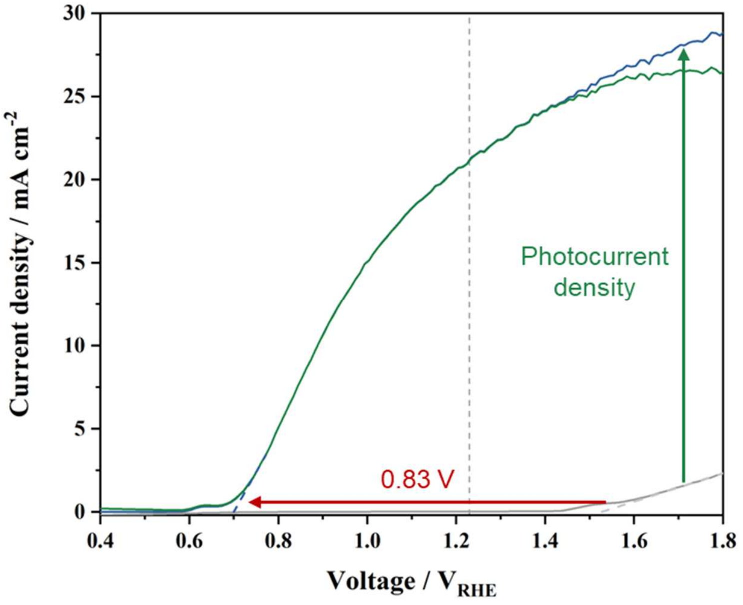

Supplementary Fig. 6 | Current–voltage scans of PM6:D18:L8-BO organic IPV-anodes in a PEC cell between +0.4 and +1.8 V_{RHE}. The difference between the polarization scans under dark condition (solid grey line) and 1 sun continuous illumination (solid blue line) shows saturation of the generated photocurrent (solid green line). Dashed lines (blue and grey) show the linear fitting used to determine the photocurrent onset potentials, which shifts by 0.83 V as indicated by a red arrow. The measurements were performed in aqueous 1 M NaOH electrolyte at 50 mV s^{-1} scan rate.

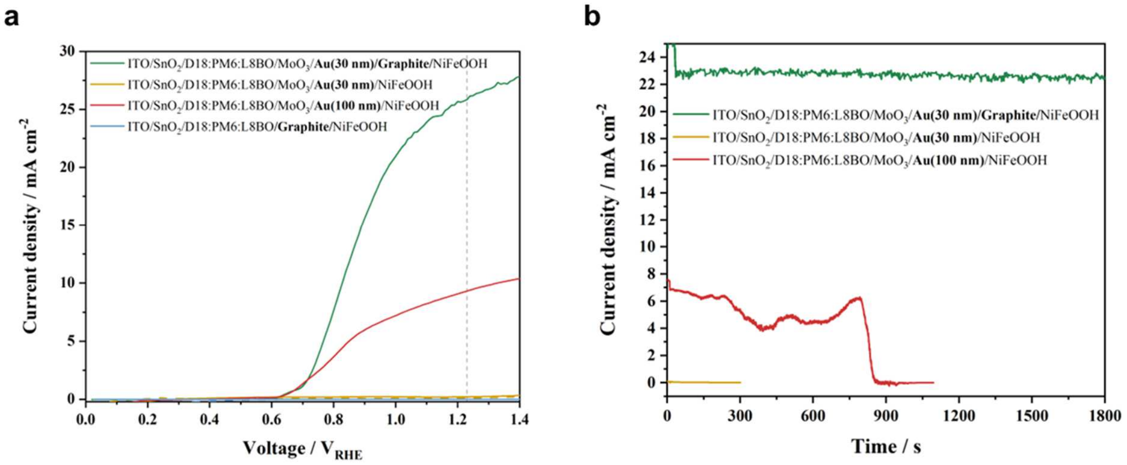

Supplementary Fig. 7 | Characterization of PM6:D18:L8-BO organic IPV-anodes in a PEC cell, with and without functionalized graphite sheet and applying different thicknesses of Au layer (0, 30, 100 nm). a, Current–voltage scans at 50 mV s^{-1} scan rate under continuous 1 sun illumination. **b,** Operational solar water oxidation stability at +1.23 V_{RHE} of the same organic IPV-anodes. The measurements were performed in aqueous 1 M NaOH electrolyte.

9. Analysis of recombination of organic solar cells with and without graphite sheet

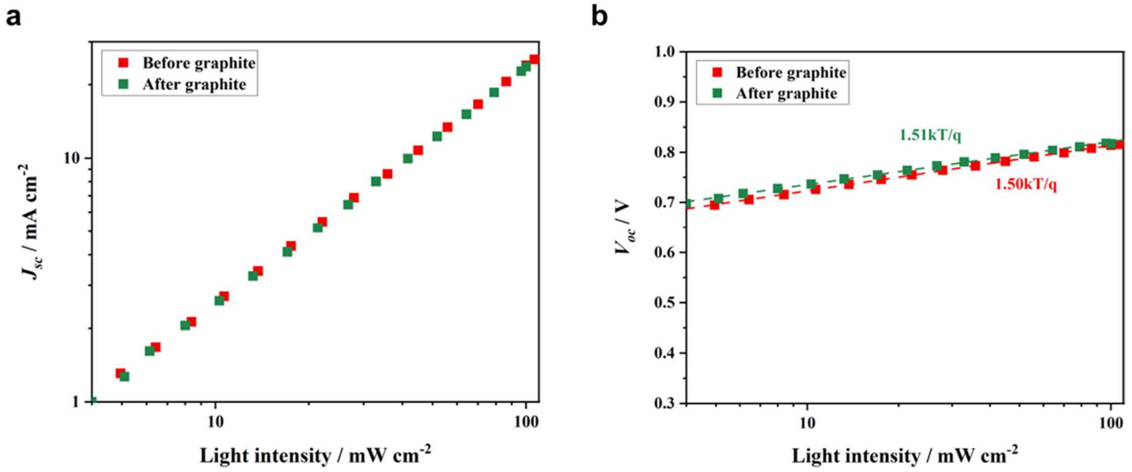

Supplementary Fig. 8 | Light intensity dependent V_{oc} and J_{sc} of organic solar cells before and after deposition of the top graphite sheet. a,b, Light intensity dependent J_{sc} (**a**), and V_{oc} (**b**) with calculated ideality factors in terms of kT/q, both showing very little change with and without the graphite protection layer.

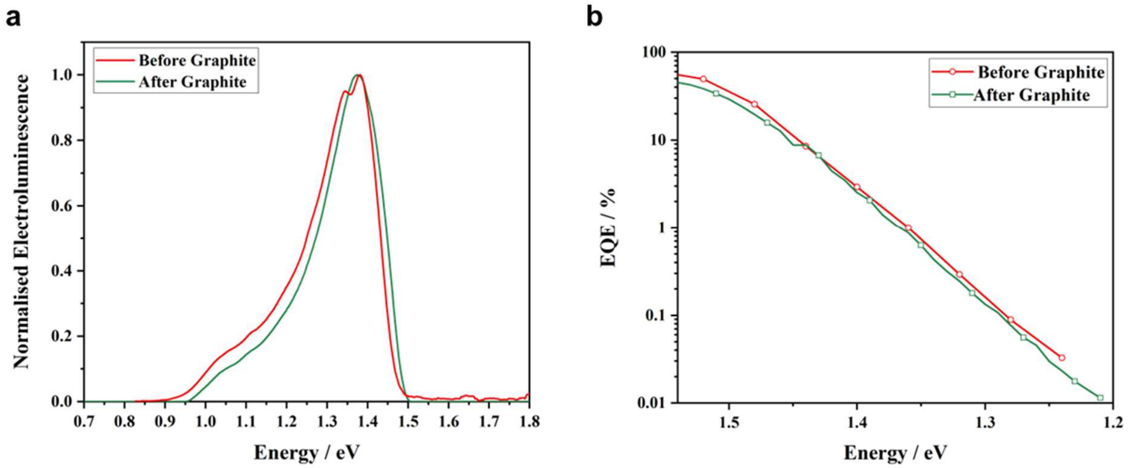

Supplementary Fig. 9 | Electroluminescence characterization PM6:D18:L8-BO organic IPV-anodes before and after depositing the top graphite sheet. a, Normalized electroluminescence spectra measured at 25 mAcm^{-2} injection current for both devices and **b,** the low energy tail of the corresponding external quantum efficiency (EQE) spectra.

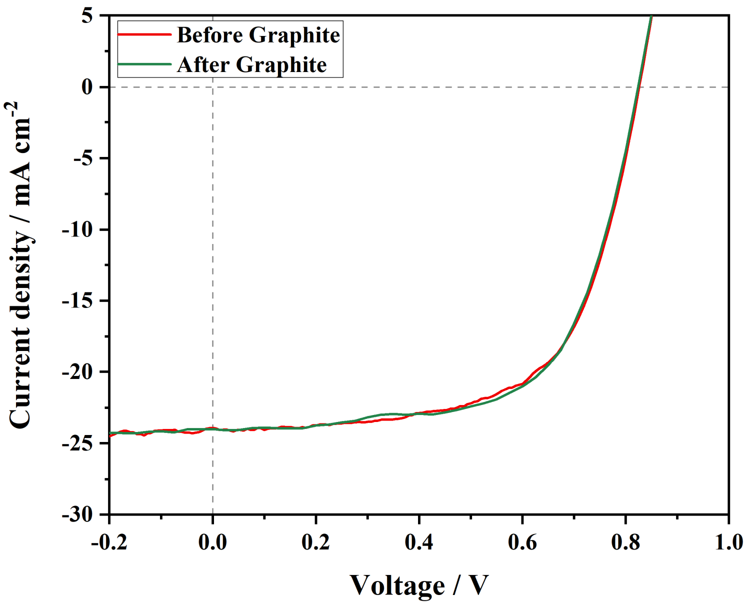

Supplementary Fig. 10 | Photovoltaic performance of an average PM6:D18:L8-BO organic IPV-anode measured as solar cells with 0.28 cm^2 active area. Current–voltage scans at 40 mV s^{-1} scan rate under 1 sun illumination before and after deposition of the top graphite sheet.

10. Voltage-loss analysis of organic solar cells

Supplementary Table 1 | Performance of a D18:PM6:L8-BO solar cell before (w/o) and after (w/) the deposition of a graphite protective layer. The radiative limit ($V_{oc,rad}$) and corresponding non-radiative voltage losses ($\Delta V_{oc,nrad}$) were calculated by using EL and EQE spectra and the reciprocity relation, as previously described.[65]

	J_{sc} (mA cm^{-2})	FF	V_{oc} (V)	$V_{oc,rad}$ (V)	$\Delta V_{oc,nrad}$ (V)
W/o graphite	27.5 ± 0.5	0.62 ± 0.02	0.83 ± 0.03	1.14 ± 0.02	0.31 ± 0.03
W/ graphite	27.0 ± 0.1	0.61 ± 0.05	0.83 ± 0.03	1.14 ± 0.04	0.31 ± 0.05

11. Analysis of solar cell performance and its distribution

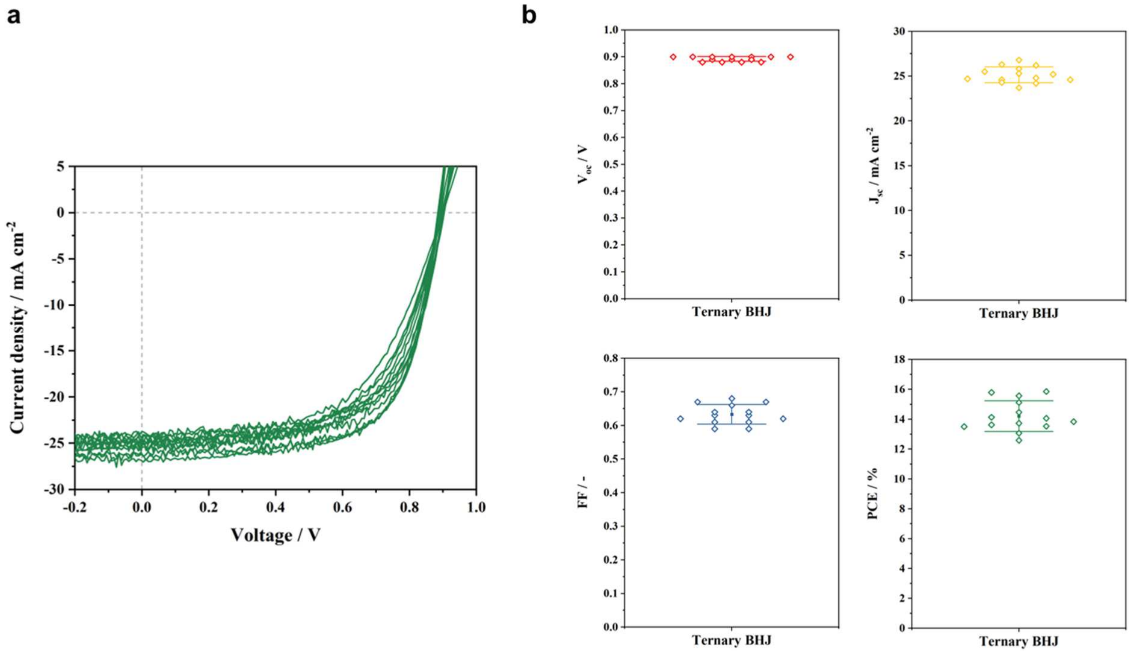

Supplementary Fig. 11 | Performance distribution of 14 PM6:D18:L8-BO organic solar cells. a, Current-voltage scans (40 mV s^{-1} scan rate) under 1 sun illumination. **b,** Distribution of V_{oc}, J_{sc}, FF and PCE values extracted from the current-voltage curves for the 14 solar cells. Open diamond symbols show the values measured for the individual devices (lateral displacement is only for clarity), the solid squares are the mean values, and the whiskers represent one standard deviation. The active area of the solar cells was 0.05 cm^2.

12. Analysis of organic IPV-anode performance and its distribution

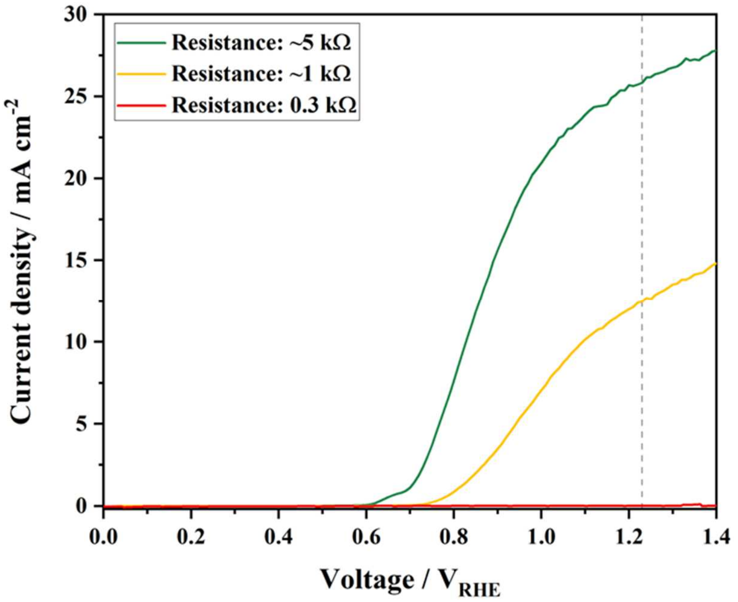

Supplementary Fig. 12 | Current-voltage scans at 50 mV s^{-1} scan rate under 1 sun illumination measured in a PEC cell for PM6:D18:L8-BO organic IPV-anodes with different shunt resistance measured between the ITO and the top graphite sheet. The ~5 kΩ resistance was achieved with a thicker (~200 nm) photoactive layer thickness, while the ~1 and 0.3 kΩ resistances were measured on devices with a thinner (110 nm) layer. Dashed vertical line indicates the applied potential of +1.23 V_{RHE}. The measurements were performed in aqueous 1 M NaOH electrolyte at 50 mV s^{-1} scan rate.

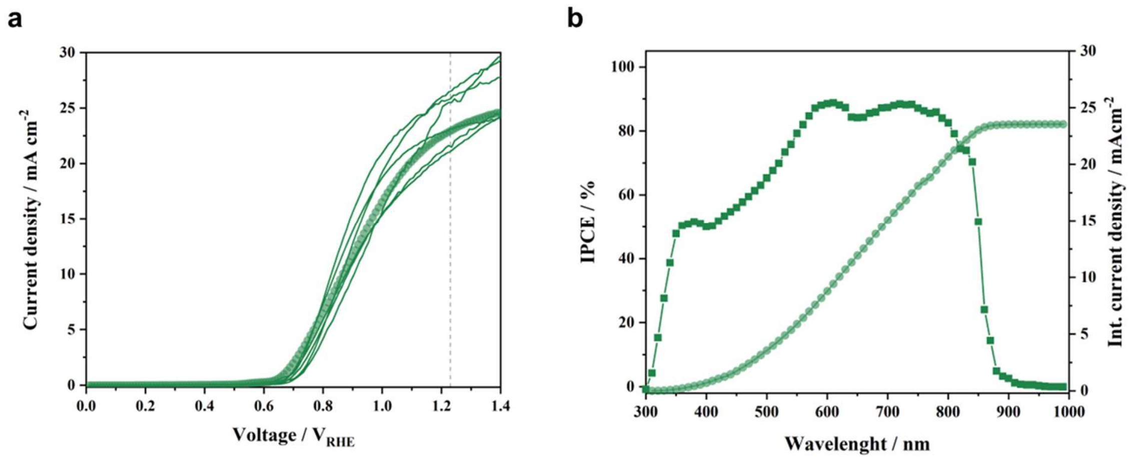

Supplementary Fig. 13 | Distribution of performance of PM6:D18:L8-BO organic IPV-anodes in a PEC cell. a, Current-voltage scans at 50 mV s^{-1} scan rate under continuous 1 sun illumination for 7 devices from different batches. Dashed vertical line indicates the applied potential of +1.23 V_{RHE}. Green circles highlight the IPV-anode measured also for IPCE. **b,** Corresponding IPCE spectrum and integrated photocurrent density at +1.23 V_{RHE} of an average organic IPV-anode. The measurements were performed in aqueous 1 M NaOH electrolyte.

13. Polymer:polymer IPV-anodes

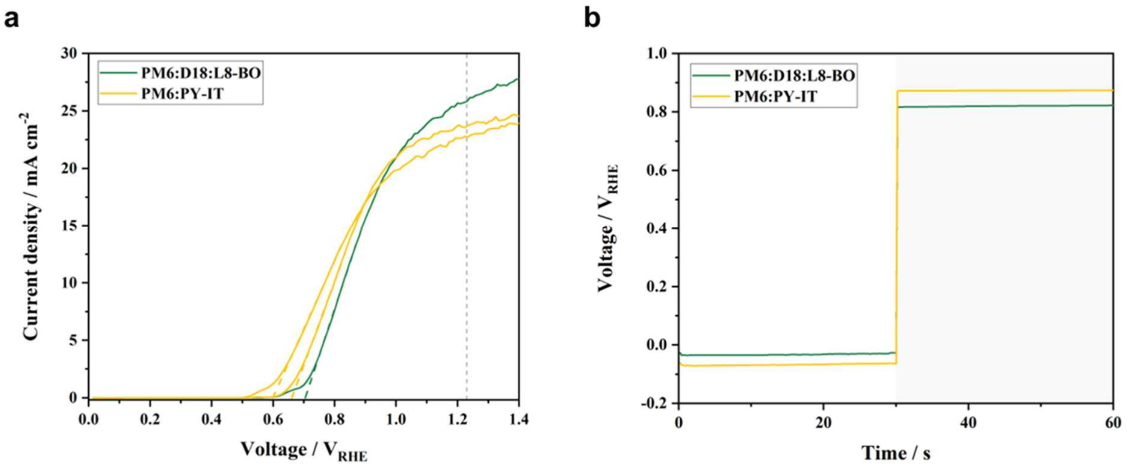

Supplementary Fig. 14 | Performance of organic IPV-anodes in a PEC cell with different photoactive layers. a, Current-voltage scans at 50 mV s^{-1} scan rate under continuous 1 sun illumination for an organic IPV-anode containing ternary PM6:D18:L8-BO compared to an all-polymer PM6:PY-IT organic photoactive layer. **b,** Open circuit potential measurements under 1 sun illumination (0 – 30 s) and in dark (30 – 60 s) of the same devices. SnO_2 electron transport layer and MoO_3 as hole transport layer was applied in all cases. Dashed vertical line indicates the standard oxidation potential of water to oxygen at +1.23 V_{RHE}. The measurements were performed in aqueous 1 M NaOH electrolyte.

14. Measurements of IPV-anodes in PEC cells with and without hole scavengers

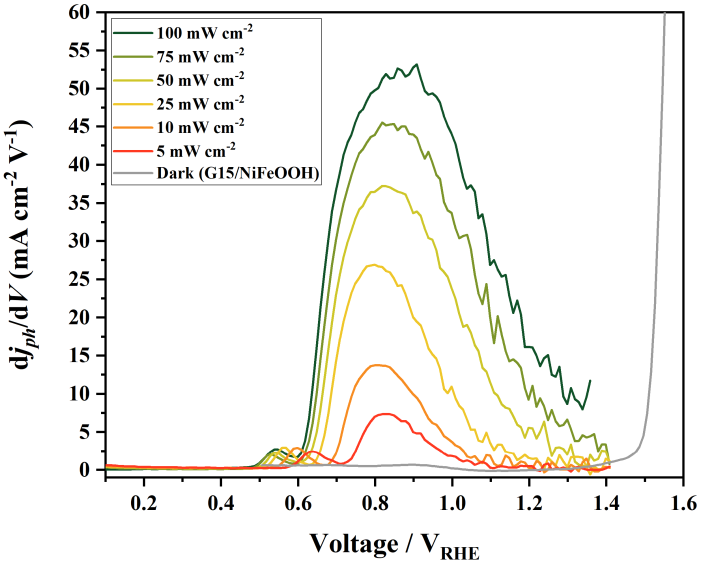

Supplementary Fig. 15 | Light intensity dependence of the first order derivative of (photo)current density as a function of voltage for a PM6:D18:L8-BO organic IPV-anode and a reference 150 µm thick NiFeOOH-functionalized graphite sheet . The measurements were performed measured in aqueous 1 M NaOH electrolyte at 50 mV s^{-1} scan rate in a PEC cell.

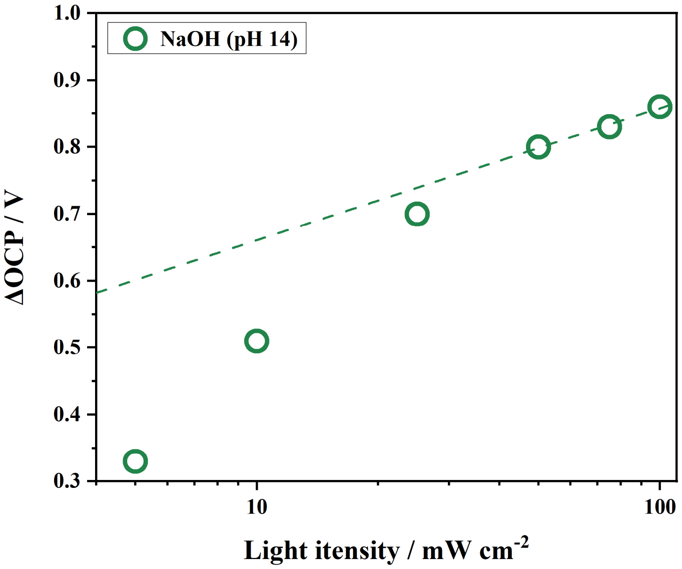

Supplementary Fig. 16 | Light intensity dependent change in OCP values upon switching off illumination of PM6:D18:L8-BO IPV-anodes measured in aqueous 1 M NaOH in a PEC cell.

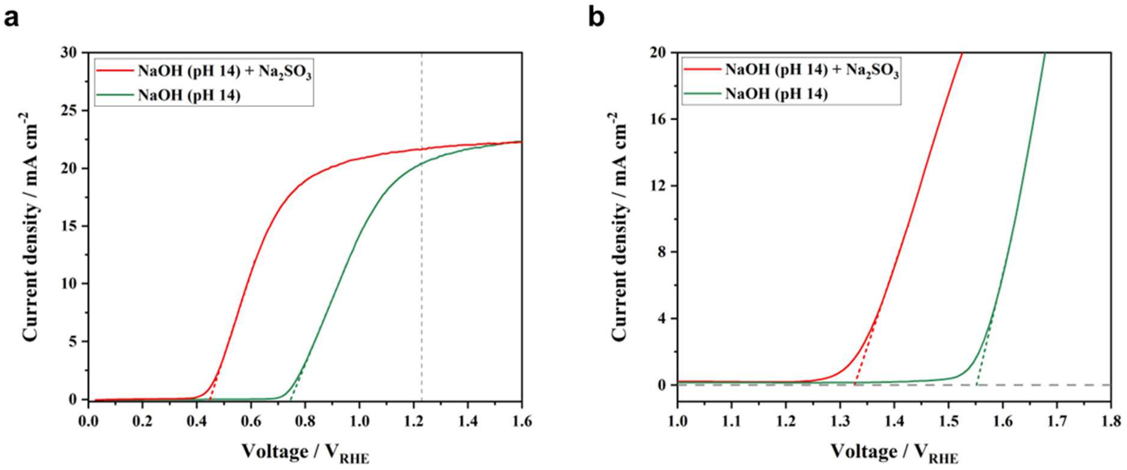

Supplementary Fig. 17 | Characterization of organic IPV-anodes and separate catalytic sheets in a (photo)electrochemical cell with and without Na_2SO_3 hole scavenger. a, Current–voltage scans under 1 sun continuous illumination for a PM6:D18:L8-BO IPV-anode. **b,** Current–voltage scans for the same graphite sheets as applied in the IPV-anodes but deposited in this case separately on glass. The onset potential of oxidation is estimated by linear fit of the current rise (dashed lines). The measurements were performed at a scan rate of 50 mV s^{-1}, in aqueous 1 M NaOH electrolyte with and without the addition of Na_2SO_3 (0.2 M).

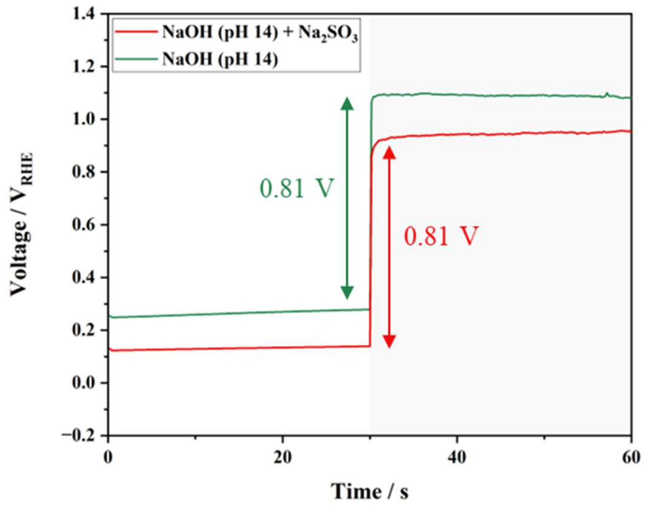

Supplementary Fig. 18 | Open circuit potential measurements in a PEC cell under 1 sun illumination (0 – 30 s) and in dark (30 – 60 s) of a PM6:D18:L8-BO single junction organic IPV-anode. Colored arrows indicate the magnitude of change in OCP upon switching off the illumination. The measurements were performed in aqueous 1 M NaOH electrolyte with and without the addition of Na_2SO_3 (0.2 M).

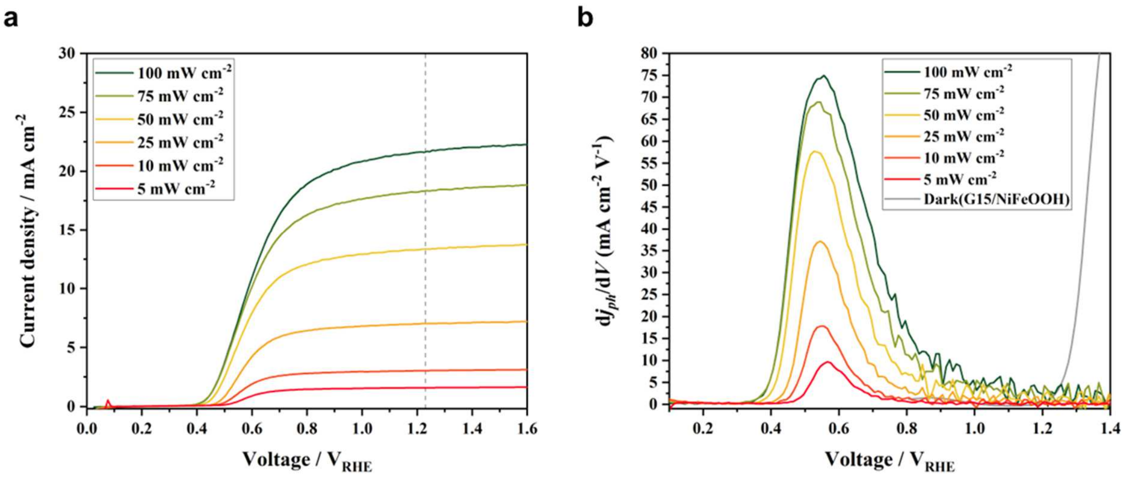

Supplementary Fig. 19 | Light intensity dependent performance of PM6:D18:L8-BO IPV-anodes with Na_2SO_3 hole scavenger measured in a PEC cell. a, Current–voltage scans of a PM6:D18:L8-BO IPV-anode under continuous illumination at different light intensities measured in aqueous 1 M NaOH electrolyte with the addition of Na_2SO_3 (0.2 M) at 50 mV s^{-1} scan rate. **b,** First order derivative of (photo)current density as a function of voltage for the organic IPV-anode and a reference 150 µm thick NiFeOOH-functionalized graphite sheet.

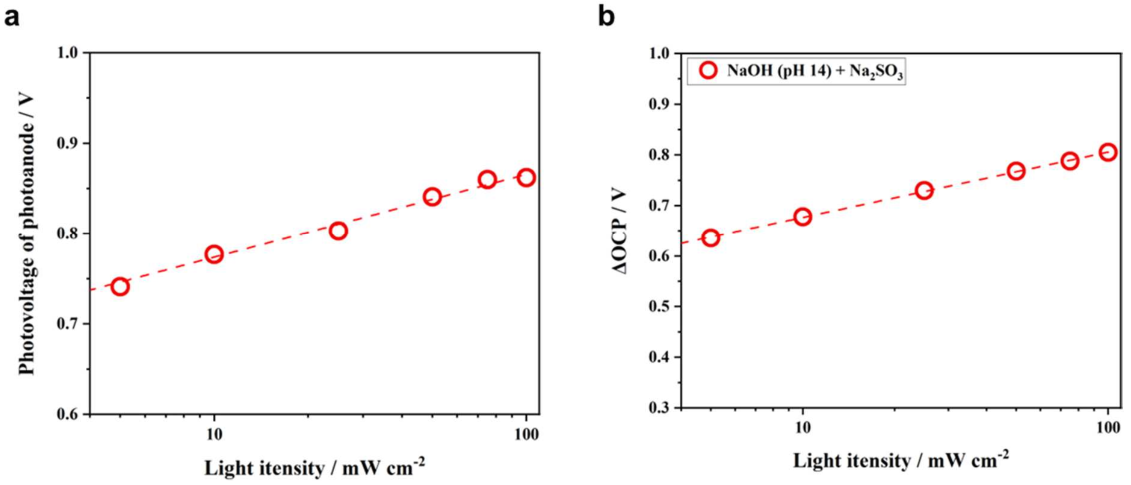

Supplementary Fig. 20 | Light intensity dependent change of photovoltage of PM6:D18:L8-BO IPV-anodes and ΔOCP in the presence of Na_2SO_3 hole scavenger measured in a PEC cell. a, Photovoltage of organic IPV-anode calculated from the onset potential shift under illumination by different light intensities compared to the dark current–voltage scan of the reference catalytic sheet. **b,** Change in OCP (vs. RHE) values upon switching off illumination of different light intensities. The measurements were performed in aqueous 1 M NaOH with the addition of Na_2SO_3 (0.2 M).

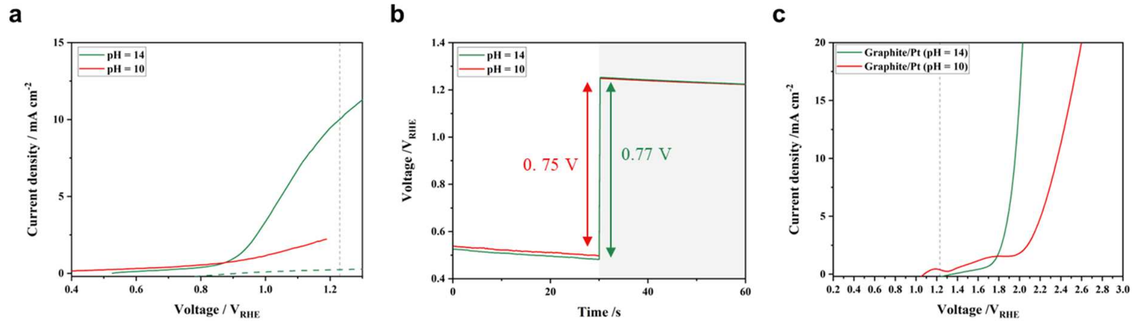

Supplementary Fig. 21 | Characterization of organic IPV-anodes in a PEC cell at different pH values applying a Pt-functionalized graphite sheet. a, Current–voltage scans under 1 sun continuous illumination for a PM6:D18:L8-BO IPV-anode. **b**, Open circuit potential measurements under 1 sun illumination (0 – 30 s) and in dark (30 – 60 s) of the organic IPV-anode at different pH values. Colored arrows indicate the magnitude of change in OCP upon switching off the illumination **c,** Current–voltage scans of the Pt-functionalized graphite sheets deposited in on glass substrate. The measurements were performed at a scan rate of 50 mV s^{-1}, in either aqueous NaOH electrolyte at pH 14 and at pH 10. The latter one was complemented with 0.2 M Na_2SO_4 to increase ionic conductivity.

15. Stability of organic solar cells and photoelectrodes at ambient environment

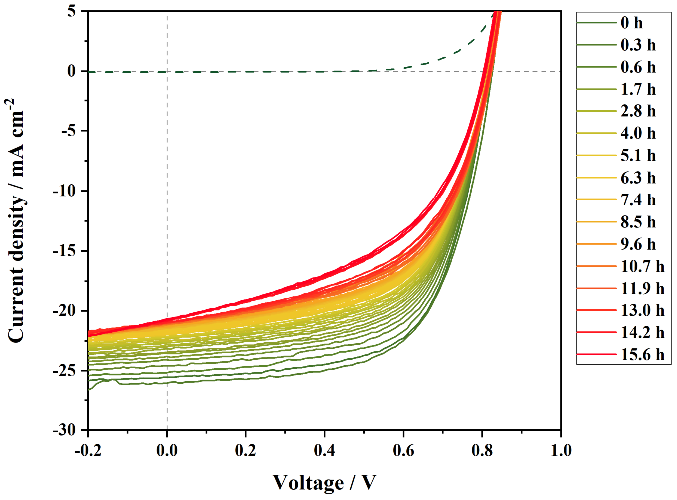

Supplementary Fig. 22 | Stability of a PM6:D18:L8-BO organic IPV-anode measured as solar cell at ambient condition, under continuous 1 sun illumination for 16 h. Current-voltage scans at 10 mV s^{-1} scan rate were recorded every 15 min, and the device was kept at 0 V applied bias between the scans. The dashed line shows the dark current-voltage scan at the beginning of the stability test.

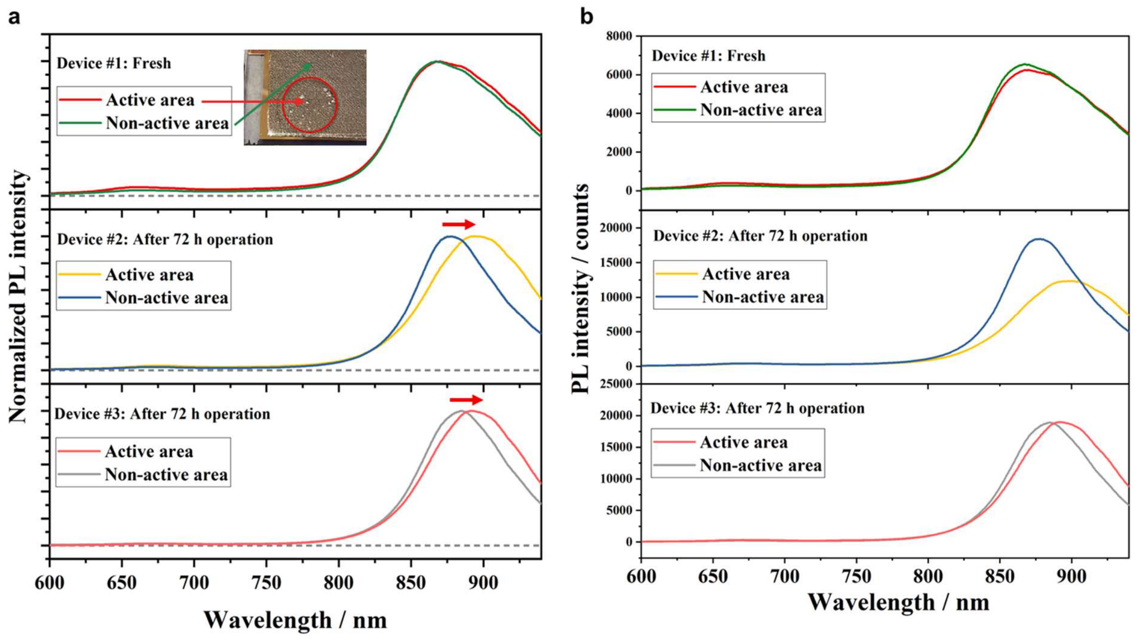

Supplementary Fig. 23 | PL spectra of the PM6:D18:L8-BO photoactive layer of three separate organic IPV-anodes. a,b, Normalized (**a**) and raw PL spectra (**b**) of the same devices. One full device was measured as fresh and two after 72 h continuous operation for PEC water oxidation under 1 sun illumination. Red arrows indicate the shift in peak position between the PL spectra recorded within the 0.28 cm^2 active area (illuminated under operation) and outside of the active area (no illumination under operation). The inset shows the front side a full device indicating the active area that is illuminated (from back) and in contact with the electrolyte (from front) during operation.

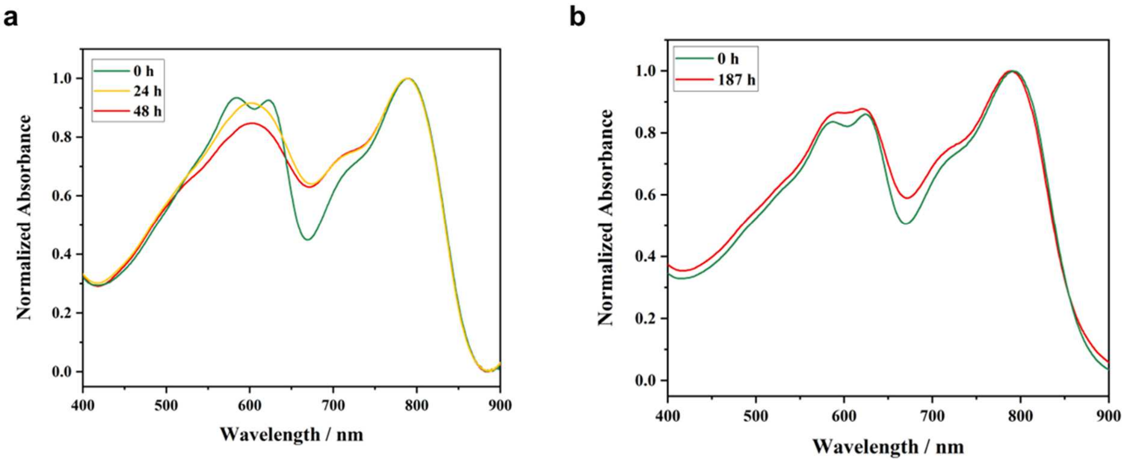

Supplementary Fig. 24 | Normalized absorbance spectra of PM6:D18:L8-BO photoactive layers before and after continuous 1 sun illumination for different time intervals. a,b, The spectra were recorded at ambient conditions for samples deposited on ITO/SnO_2 substrates (**a**) and under nitrogen (**b**) for samples deposited on quartz substrates.

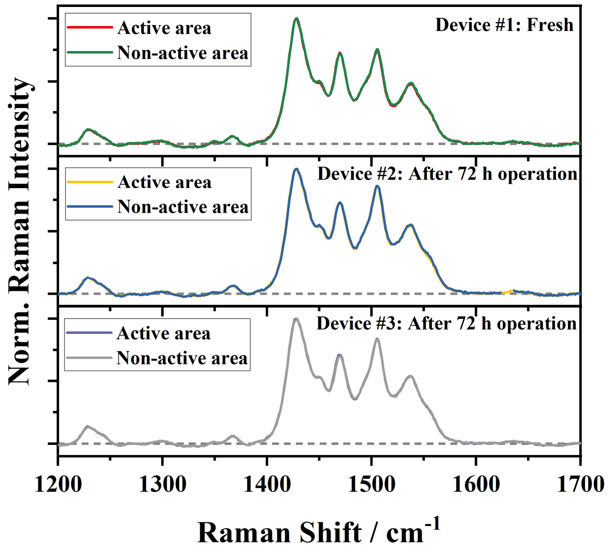

Supplementary Fig. 25 | Normalized Raman spectra of the PM6:D18:L8-BO photoactive layer of three separate organic IPV-anodes. One full device was measured as fresh and two after 72 h continuous operation for PEC water splitting under 1 sun illumination. The Raman spectra were recorded within the 0.28 cm^2 active area (illuminated under operation) and outside of the active area (no illumination under operation).

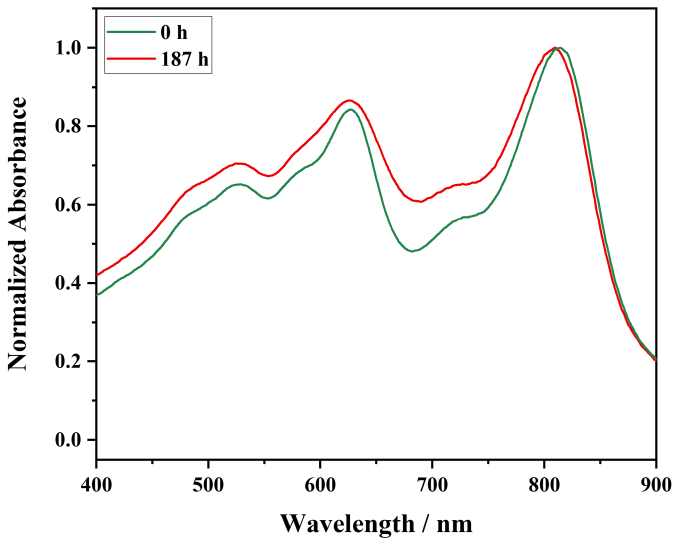

Supplementary Fig. 26 | Normalized absorbance spectra of PM6:PY-IT photoactive layers before and after continuous 1 sun illumination for different time intervals. The spectra were recorded for samples deposited on quartz substrates and kept under nitrogen. Signatures of degradation are the slight loss of vibronic structure and apparent broadening of the polymer peaks consistent with changes observed in the ternary blend.

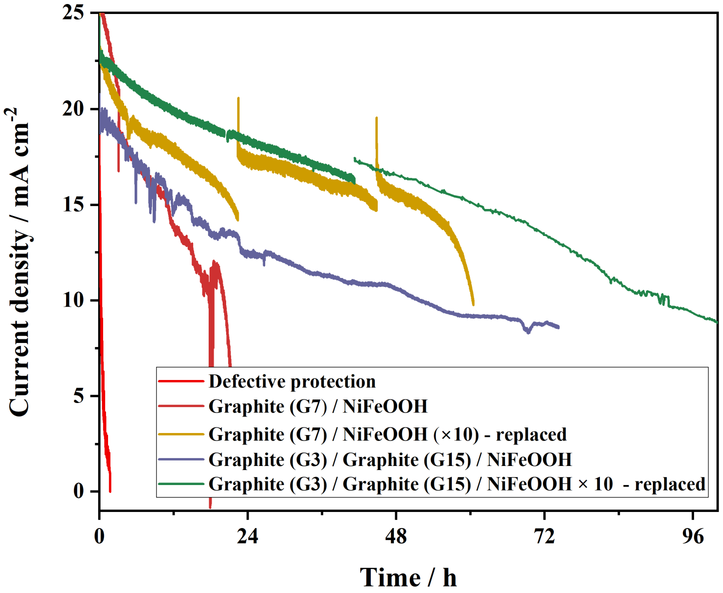

Supplementary Fig. 27 | Operational water-oxidation stability at +1.23 V_{RHE} of organic IPV-anodes measured in a PEC cell with different graphite sheets at ambient conditions. Photocurrent density decay comparison for organic IPV-anodes with defective graphite sheet (electrolyte could penetrate the photoactive layer at the edges, red line) with a single 70 µm thick graphite sheet (G7, dark red and brown line) and with a combination of a denser, 25 µm thick graphite sheet (G3) and a more porous 150 µm thick graphite sheet (G15, purple line and green line). There was a 420 nm cut-off UV filter used in all cases, except for the first 3 h of the single G7 protected sample (dark red line). In some cases (brown and green lines) fresh catalyst-functionalized graphite sheet was added after ~20 h and ~40 h operation. The graphite sheets were functionalized by electrodeposited NiFeOOH, in some cases with a ×10 thicker layer. All stability measurements were performed in an aqueous 1 M NaOH electrolyte under 1 sun illumination.

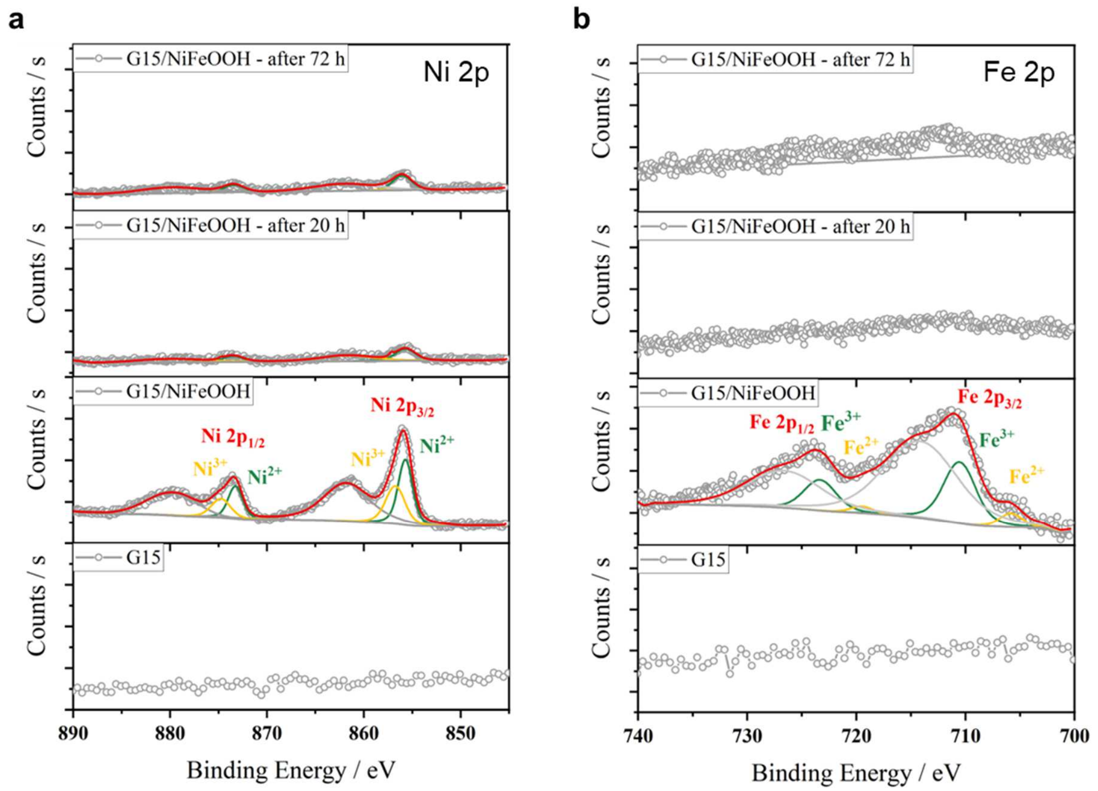

Supplementary Fig. 28 | High-resolution deconvoluted XPS spectra recorded at the surface of a 150 µm graphite sheet without and with a thinner layer or electrodeposited NiFeOOH and before after 20 and 72 h of water oxidation operation. a,b, XPS spectra of Ni 2p (**a**) and Fe 2p (**b**). Grey empty circles show the raw data, red lines the cumulative fits, while grey lines indicate the background used for the fits. The water oxidation stability measurement of the graphite sheets deposited on glass was performed in an aqueous 1 M NaOH electrolyte at 20 mA cm^{-2} current density.

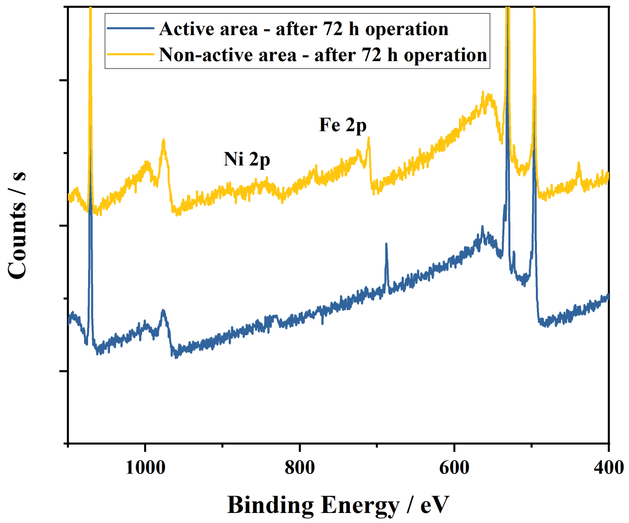

Supplementary Fig. 29 | XPS survey spectra recorded within the active area (in contact with the electrolyte) and outside of the active area (not in contact with the electrolyte) at the surface of an organic IPV-anode that was under continuous operation for solar water oxidation for 72 h in an aqueous 1 M NaOH.

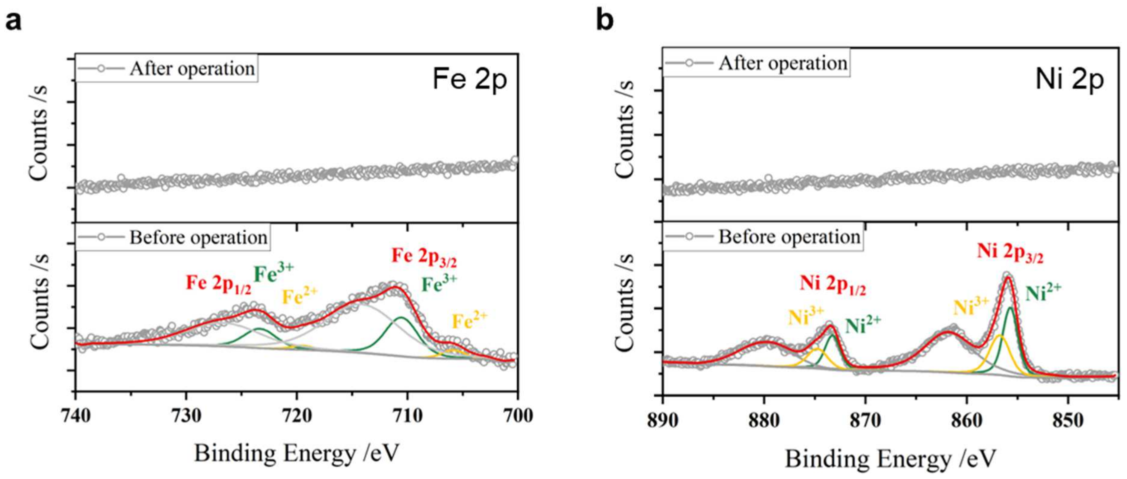

Supplementary Fig. 30 | High-resolution deconvoluted XPS spectra recorded at the surface of organic IPV-anodes. a,b, XPS spectra of Ni 2p (**a**) and Fe 2p (**b**). The spectra were measured for devices after days-long continuous operation for solar water oxidation in an aqueous 1 M NaOH electrolyte, and for a fresh sample before operation.

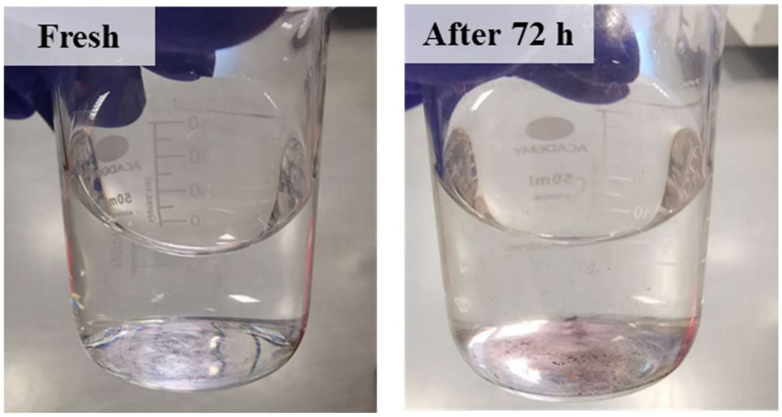

Supplementary Fig. 31 | Photographs of the aqueous, 1 M NaOH electrolytes before and after continuous 72 h water oxidation operation of a graphite sheet (150 µm) functionalized by NiFeOOH at 20 mA cm^{-2} current density.

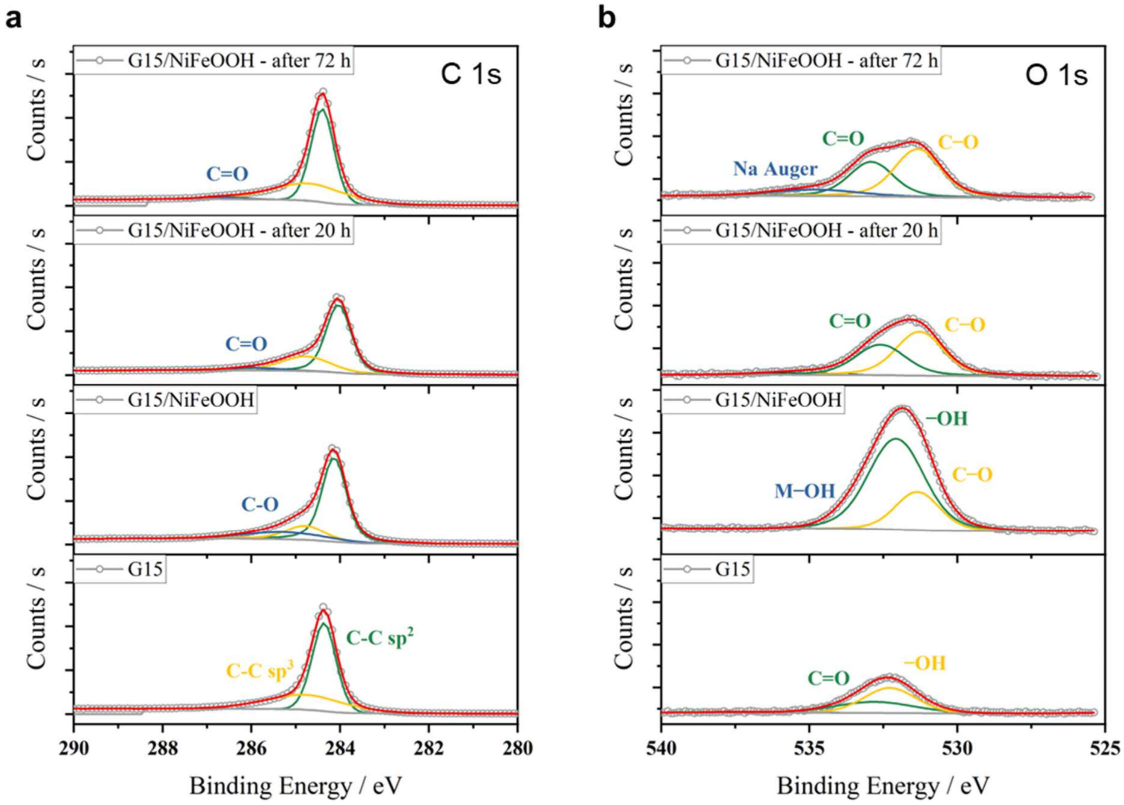

Supplementary Fig. 32 | High-resolution deconvoluted XPS spectra recorded at the surface of a 150 µm graphite sheet without and with a thinner layer of electrodeposited NiFeOOH and before after 20 and 72 h of water oxidation operation. a,b, XPS spectra of C 1s (**a**) and O 1s (**b**). Grey empty circles show the raw data, red lines the cumulative fits, while grey lines indicate the background. The water oxidation stability measurement of the graphite sheets deposited on glass was performed in an aqueous 1 M NaOH electrolyte at 20 mA cm^{-2} current density.

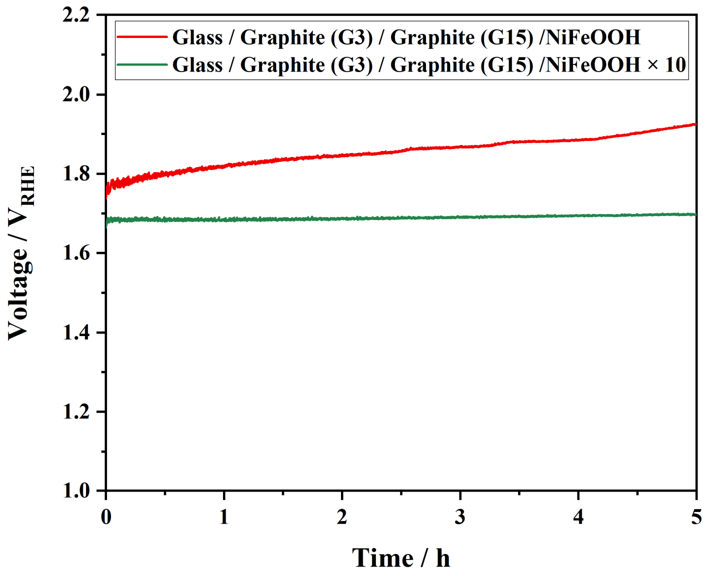

Supplementary Fig. 33 | Operational stability of graphite catalytic sheet at 20 mA cm^{-2} current density in dark with a thinner and thicker electrodeposited NiFeOOH electrocatalyst layer. The 150 µm thick graphite sheets (G15) were attached to glass slides previously covered by 30 µm thick, dense graphite sheets (G3) and only the top surface was available for water oxidation, as in the IPV-anodes. The measurements were performed at pH 14 in an aqueous 1 M NaOH electrolyte.

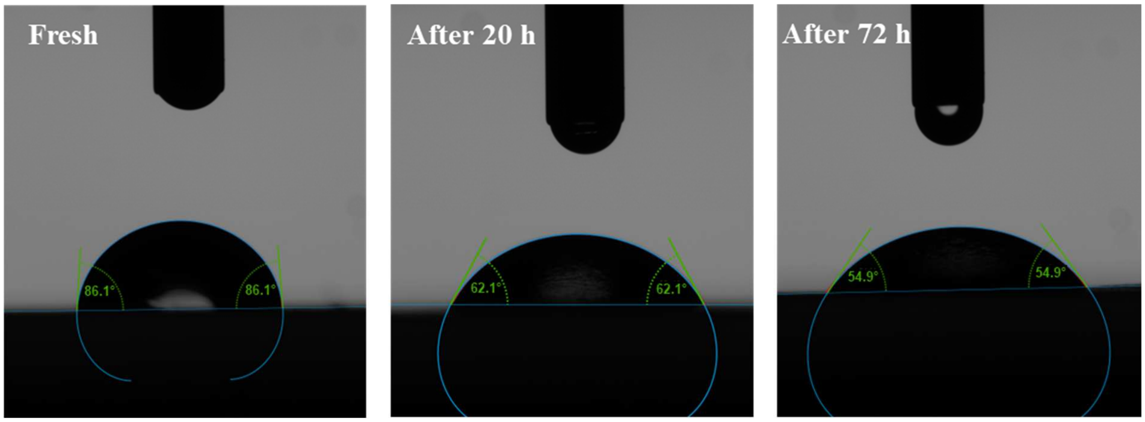

Supplementary Fig. 34 | Water contact angle measurements on catalytic graphite sheets before and after continuous water oxidation operation. The water oxidation stability measurement of the graphite sheets (150 µm thick) functionalized by a thinner layer of NiFeOOH and deposited on glass was performed in an aqueous 1 M NaOH electrolyte at 20 mA cm^{-2} current density.

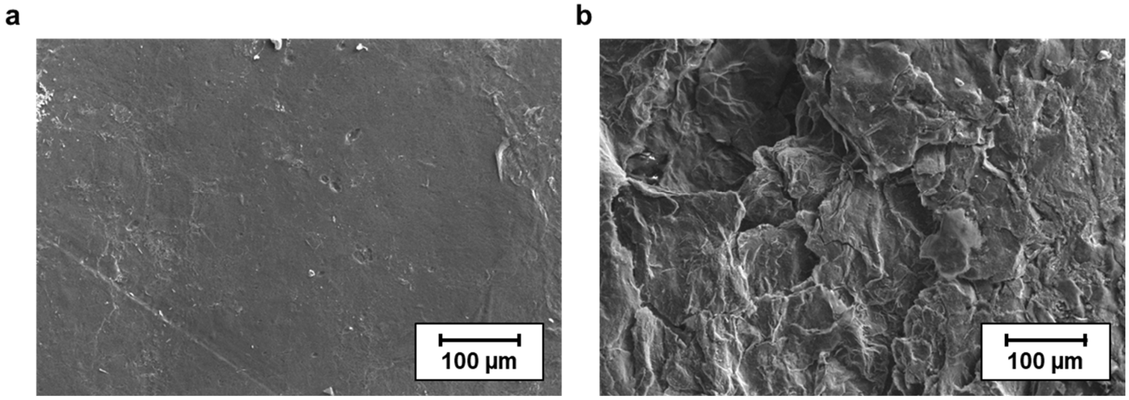

Supplementary Fig. 35 | Morphology of graphite sheet before and after stability test. a,b, Scanning electron micrographs of a NiFeOOH-functionalized 150 µm thick graphite sheet before (**a**) and after (**b**) 72 h operational PEC stability measurement of the organic IPV-anode. The stability measurement of the full IPV-anode was performed in an aqueous 1 M NaOH electrolyte under 1 sun illumination at +1.23 V_{RHE}.

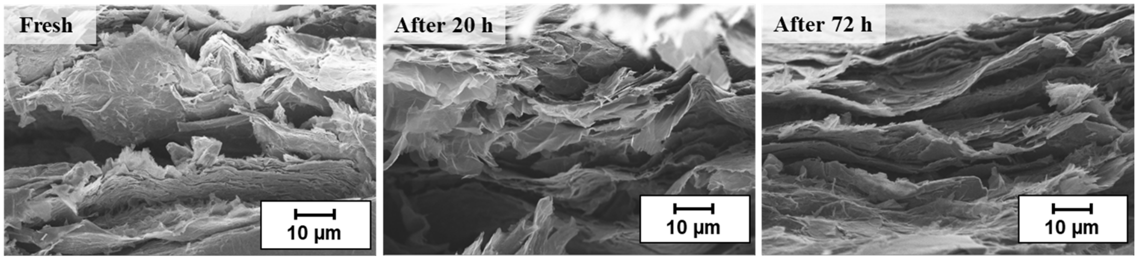

Supplementary Fig. 36 | Cross-section scanning electron micrographs of graphite sheets before and after continuous water oxidation operation. The water oxidation stability measurement of the graphite sheets (150 µm thick) functionalized by a thinner layer or NiFeOOH and deposited on glass was performed in an aqueous 1 M NaOH electrolyte at 20 mA cm^{-2} current density.

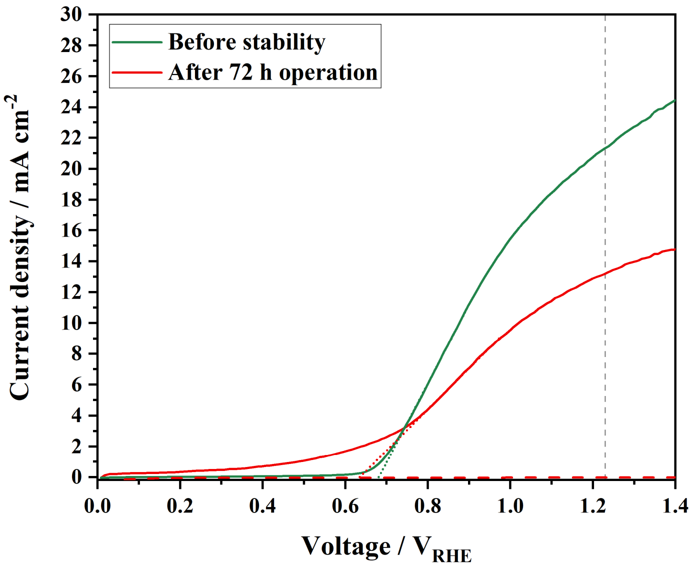

Supplementary Fig. 37 | Performance of organic IPV-anode measured in a PEC cell before and after 72 h operational stability. Dotted lines show the linear fits used to determine the photocurrent onset potential. Dashed vertical line indicates the standard oxidation potential of water to oxygen at +1.23 V_{RHE}. The current-voltage scans were recorded under 1 sun illumination in aqueous 1 M NaOH electrolyte at 50 mV s^{-1} scan rate.

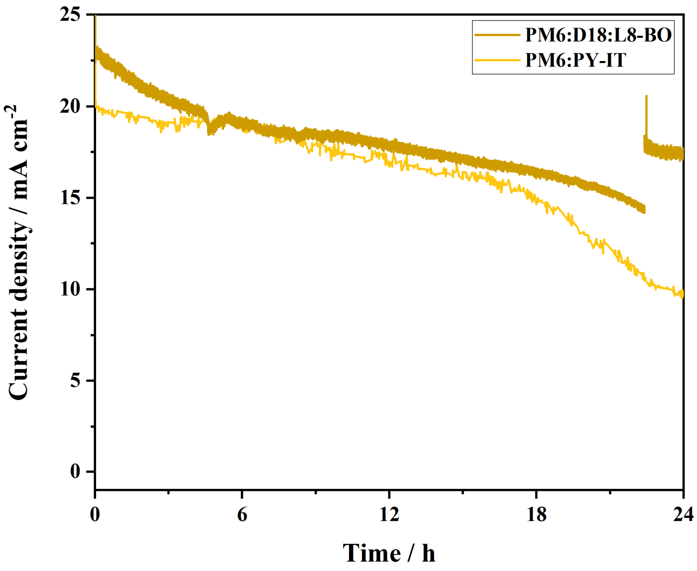

Supplementary Fig. 38 | Operational water-oxidation stability measured in PEC cell at +1.23 V_{RHE} for IPV-anodes with PM6:D18:L8-BO photoactive layer compared to PM6:PY-IT. The devices contained 70 µm thick graphite sheet functionalized by electrodeposited NiFeOOH. The measurements were performed in an aqueous 1 M NaOH electrolyte under 1 sun illumination.

16. Tandem organic IPV-anodes and solar cells

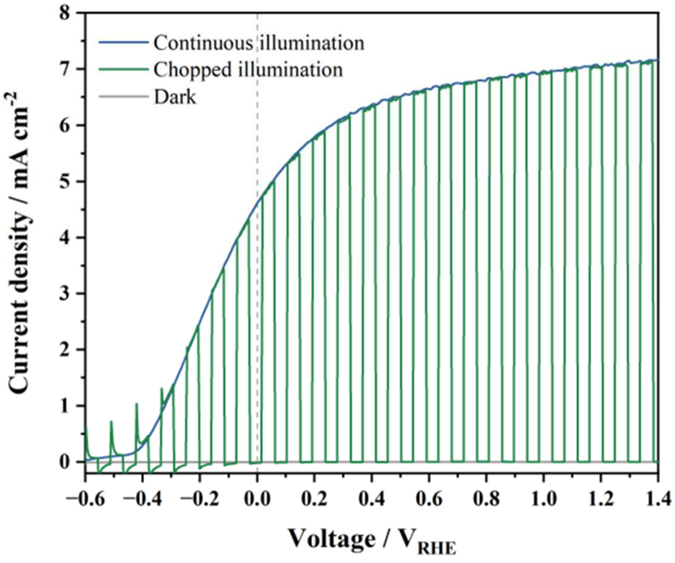

Supplementary Fig. 39 | Three-electrode water-oxidation performance of the monolithic organic tandem IPV-anode measured in PEC cell with PM6:D18:L8-BO and PTQ10:GS-ISO absorber layers. Current-voltage scans at 50 mV s^{-1} scan rate in dark, under 1 sun continuous and chopped illumination for the monolithic tandem IPV-anode. The scan rate for the chopped illumination was 20 mV s^{-1}. The dashed vertical line indicates the standard potential of water reduction to hydrogen at 0 V_{RHE}. The measurements were performed in aqueous 1 M NaOH electrolyte.

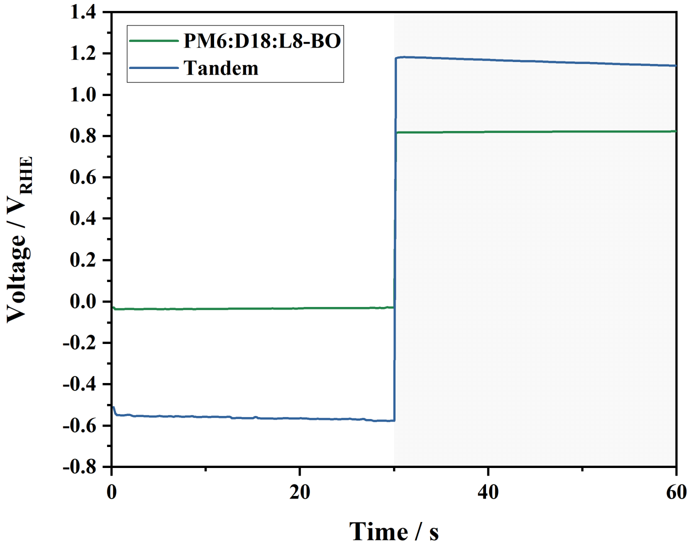

Supplementary Fig. 40 | Open circuit potential measurements in PEC cell under 1 sun illumination (0 – 30 s) and in dark (30 – 60 s) of the ternary single junction and organic tandem IPV-anodes. The tandem IPV-anode contained the same narrow-bandgap PM6:D18:L8-BO active layer as the single junction device with the addition of the wide-bandgap PTQ10:GS-ISO absorber layer. The measurements were performed in aqueous 1 M NaOH electrolyte.

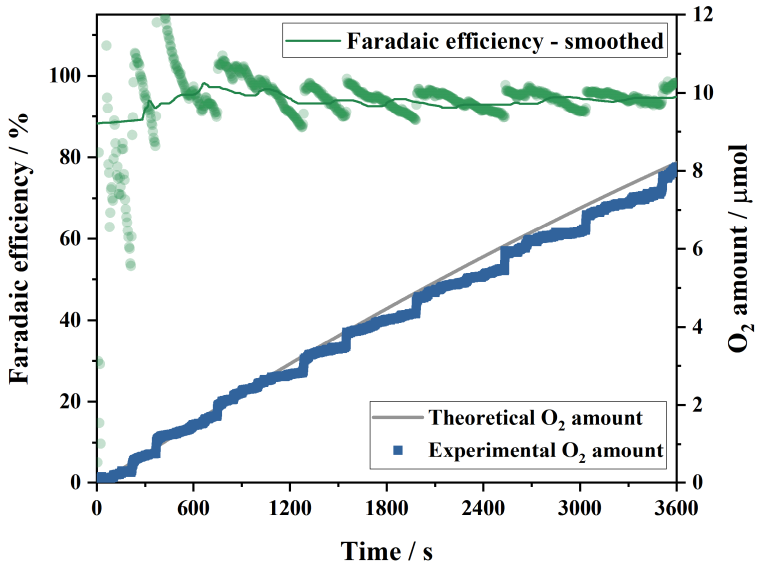

Supplementary Fig. 41 | Faradaic efficiency of a tandem organic IPV-anode. The value of Faradaic efficiency was calculated from the measured amount of generated O_2 compared to the theoretical amount of O_2 based on the recorded photocurrent. The measurement was performed in an aqueous 1 M NaOH electrolyte.

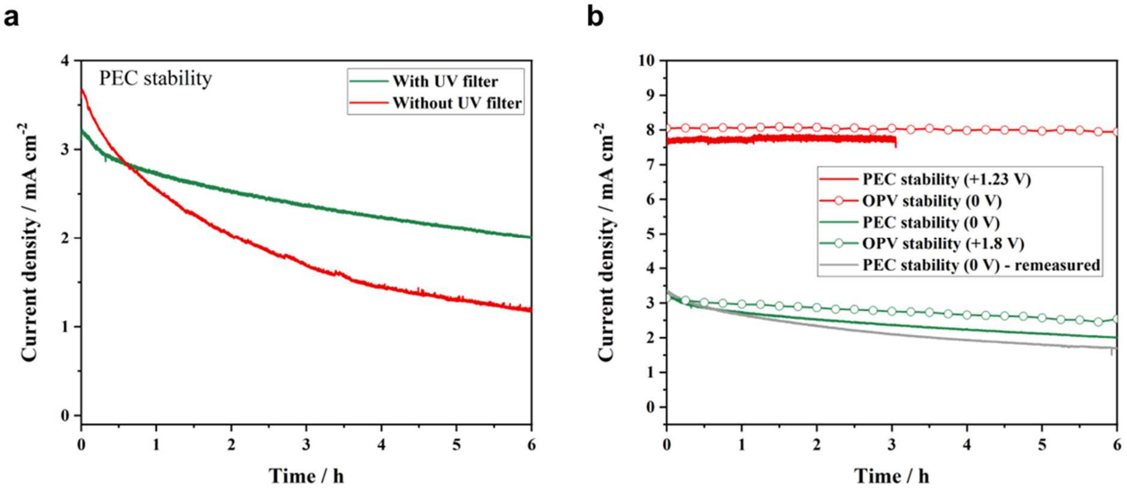

Supplementary Fig. 42 | Two-electrode, operational stability of the tandem organic IPV-anodes and solar cells. a, Unassisted, two-electrode PEC stability of a tandem organic IPV-anode with and without the application of a 420 nm cut-off UV filter. **b,** Comparison of PEC and OPV operational stability of a tandem organic IPV-anode measured at different applied voltages. One of the IPV-anode devices was re-measured (grey line) a few hours after the previous measurement (green line). The devices contained 150 µm thick graphite sheet on top of a denser, 30 µm thick graphite sheet, functionalized by a thicker layer of electrodeposited NiFeOOH. The measurements were performed in an aqueous 1 M NaOH electrolyte under 1 sun illumination.

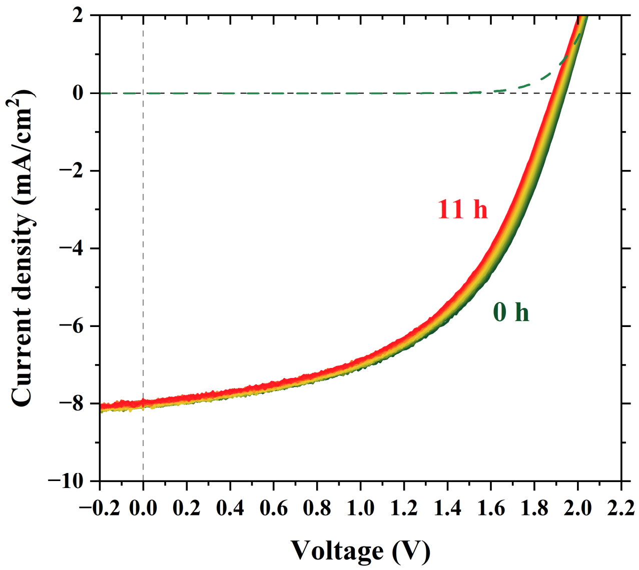

Supplementary Fig. 43 | Stability of a tandem organic IPV-anode measured as solar cell at ambient condition, under continuous 1 sun illumination for 11 h. Current-voltage scans at 10 mV s^{-1} scan rate were recorded every 15 min, and the device was kept at 0 V applied bias between the scans. The dashed line shows the dark current-voltage scan at the beginning of the stability test.

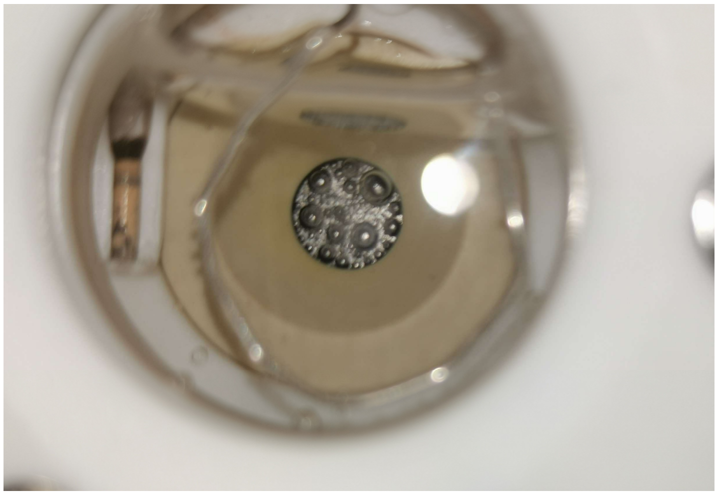

Supplementary Fig. 44 | Photograph of O_2 bubbles covering the surface of a tandem organic IPV-anode during unassisted water-splitting operation.

17. Reported solar water oxidation performance comparison of monolithic, fully integrated devices

Supplementary Table 2 | Comparison of reported single-junction photoanodes and IPV-anodes with various photoactive layers tested for solar water oxidation to oxygen (no sacrificial agents).

No.	Reference	Photoactive semiconductor layer	Layers on top of photoactive layer	Bandgap	E_{on}	j_{ph} at +1.23 V_{RHE}	Stability duration	Preserved percentage of initial j_{ph}
1	This work	Organic (PM6:D18:l8-BO)	MoO_3/Au/Graphite/NiFeOOH	1.45 eV	+0.7 V_{RHE}	26.4 mA cm^{-2}	40 h	70 %
2	This work	Organic (PM6:PY-IT)	MoO_3/Au/Graphite/NiFeOOH	1.45 eV	+0.6 V_{RHE}	23.3 mA cm^{-2}	-	-
3	Ref. 39	Organic (PTCDA/ PTCDA:PCBM)	PCBM	2.15 eV	+0.55 V_{RHE}	0.12 mA cm^{-2}	0.3 h	59 %
4	Ref. 14	Organic (PBDTTTPD: PNDITCVT)	PTAA/Li-IrO_x	1.64 eV	+0.3 V_{RHE}	2.3 mA cm^{-2}	0.5 h	43 %
5	Ref. 16	Organic (PM6:Y6)	PM6/Au/NiFeOOH	1.64 eV	+0.85 V_{RHE}	4.0 mA cm^{-2}	1 h	50 %
6	Ref. 17	Organic (PBDBT: ITIC)	GaIn/Ni/NiFe-LDH	1.65 eV	+0. 55 V_{RHE}	15.1 mA cm^{-2}	10 h	98 %
7	Ref. 40	Metal oxide (WO_3)	-	2.75 eV	+0.6 V_{RHE}	3.7 mA cm^{-2}	0.3 h	75 %
8	Ref. 41	Metal oxide (Pt: α-Fe_2O_3)	Co-Pi	2.1 eV	+0.7 V_{RHE}	4.3 mA cm^{-2}	3 h	85 %
9	Ref. 42	Metal oxide (α-Fe_2O_3)	Ag/Co-Pi	2.0 eV	+0.7 V_{RHE}	4.7 mA cm^{-2}	5 h	95 %
10	Ref. 43	Metal oxide ($BiVO_4$)	Bi/NiFeOOH	2.4 eV	+0.3 V_{RHE}	4.7 mA cm^{-2}	13 h	100 %

11	Ref. 44	Metal oxide ($BiVO_4$)	Co_3O_4/FeOOH/NiOOH	2.4 eV	+0.1 V_{RHE}	6.3 mA cm^{-2}	4 h	88 %
12	Ref. 45	Perovskite ($CsPbBr_3$)	Carbon/Graphite/Ir-based catalyst	2.3 eV	+0.55 V_{RHE}	4.5 mA cm^{-2}	27 h	80 %
13	Ref. 22	Perovskite ($CsPbBr_3$)	Carbon/Graphite/NiFeOOH	2.3 eV	+0.4 V_{RHE}	8.1 mA cm^{-2}	100 h	75 %
14	Ref. 46	Perovskite ($FAPbBr_3$)	Carbon/Graphite/NiFe alloy/NiFe LDH	2.3 eV	–0.2 V_{RHE}	8.7 mA cm^{-2}	73 h	83 %
15	Ref. 47	Perovskite ($MAPbI_3$)	Carbon/Ag/Carbon	1.6 eV	+0.95 V_{RHE}	12 mA cm^{-2}	12 h	70 %
16	Ref. 48	Perovskite ($FA_{0.83}Cs_{0.17}Pb(I_{0.8}Br_{0.2})_3$)	Spiro-OMeTAD/Au/Graphite/Ni	1.63 eV	+0.54 V_{RHE}	17.4 mA cm^{-2}	40 h	38 %
17	Ref. 49	Perovskite ($(FAPbI_3)_{0.95}(MAPbBr_3)_{0.05}$)	Spiro-OMeTAD/PTPD/Au/Ni/FM/Ni_3S_2	1.52 eV	+0.25 V_{RHE}	22.9 mA cm^{-2}	12 h	90 %
18	Ref. 50	Perovskite ($FA_{0.93}MA_{0.07}PbI_3$)	Spiro-OMeTAD/Au/Carbon/Ni/NiFe	1.52 eV	+0.56 V_{RHE}	24.3 mA cm^{-2}	48 h	50 %
19	Ref. 51	Silicon (p^+-Si)	TiO_2/Ir	1.1 eV	+0.95 V_{RHE}	15 mA cm^{-2}	24 h	100 %
20	Ref. 37	Silicon (n-Si)	SiO_x/CoO_x/NiO_x	1.1 eV	+0.9 V_{RHE}	28 mA cm^{-2}	1700 h	100 %
21	Ref. 38	Silicon (np^+-Si)	SiO_x/NiFe alloy	1.1 eV	+0.9 V_{RHE}	30.7 mA cm^{-2}	100 h	100 %
22	Ref. 52	III-V (GaAs)	TiO_2/Ni	1.42 eV	+0.67 V_{RHE}	14.3 mA cm^{-2}	25 h	85 %
23	Ref. 53	III-V (GaAs)	Pt/Ti/Pt/Au/Ti/Pt/IrO_x	1.42 eV	+0.55 V_{RHE}	12.5 mA cm^{-2}	-	-

18. Supplementary references

3. Pinaud, B. A. *et al.* Technical and economic feasibility of centralized facilities for solar hydrogen production via photocatalysis and photoelectrochemistry. *Energy Environ Sci* **6**, 1983–2002 (2013).

14. Cho, H. H. *et al.* A semiconducting polymer bulk heterojunction photoanode for solar water oxidation. *Nature Catalysis 2021 4:5* **4**, 431–438 (2021).

16. Ho Lee, T. *et al.* A Dual Functional Polymer Interlayer Enables Near-Infrared Absorbing Organic Photoanodes for Solar Water Oxidation. *Adv Energy Mater* 12, 2103698 (2022).

17. Yu, J. M. *et al.* High-performance and stable photoelectrochemical water splitting cell with organic-photoactive-layer-based photoanode. *Nat Commun* 11, 1–9 (2020).

22. Daboczi, M. *et al.* Scalable All-Inorganic Halide Perovskite Photoanodes with >100 h Operational Stability Containing Earth-Abundant Materials. *Advanced Materials* 2304350 (2023).

29. Du, C. *et al.* Hematite-Based Water Splitting with Low Turn-On Voltages. *Angewandte Chemie International Edition* **52**, 12692–12695 (2013).

30. Power, G. P. & Ritchie, I. M. Mixed potentials: Experimental illustrations of an important concept in practical electrochemistry. *J Chem Educ* **60**, 1022–1026 (1983).

36. Wang, Y. *et al.* The critical role of the donor polymer in the stability of high-performance non-fullerene acceptor organic solar cells. *Joule* **7**, 810–829 (2023).

37. Zhou, X. *et al.* Interface engineering of the photoelectrochemical performance of Ni-oxide-coated n-Si photoanodes by atomic-layer deposition of ultrathin films of cobalt oxide. *Energy Environ Sci* **8**, 2644–2649 (2015).

38. Yu, X. *et al.* NiFe Alloy Protected Silicon Photoanode for Efficient Water Splitting. *Adv Energy Mater* **7**, 1601805 (2017).

39. Liu, G., Chen, C., Ji, H., Ma, W. & Zhao, J. Photo-electrochemical water splitting system with three-layer n-type organic semiconductor film as photoanode under visible irradiation. *Sci China Chem* **55**, 1953–1958 (2012).

40. Wang, S. *et al.* Synergistic crystal facet engineering and structural control of WO3 films exhibiting unprecedented photoelectrochemical performance. *Nano Energy* **24**, 94–102 (2016).

41. Kim, J. Y. *et al.* Single-crystalline, wormlike hematite photoanodes for efficient solar water splitting. *Scientific Reports 2013 3:1* **3**, 1–8 (2013).

42. Peerakiatkhajohn, P. *et al.* Stable Hematite Nanosheet Photoanodes for Enhanced Photoelectrochemical Water Splitting. *Advanced Materials* **28**, 6405–6410 (2016).

43. Cui, J. *et al.* 2D Bismuthene as a Functional Interlayer between BiVO4 and NiFeOOH for Enhanced Oxygen-Evolution Photoanodes. *Adv Funct Mater* **32**, (2022).

44. He, B. *et al.* General and Robust Photothermal-Heating-Enabled High-Efficiency Photoelectrochemical Water Splitting. *Advanced Materials* **33**, 2004406 (2021).

45. Poli, I. *et al.* Graphite-protected CsPbBr3 perovskite photoanodes functionalised with water oxidation catalyst for oxygen evolution in water. *Nat Commun* **10**, 1–10 (2019).

46. Yang, H. *et al.* Monolithic FAPbBr3 photoanode for photoelectrochemical water oxidation with low onset-potential and enhanced stability. *Nature Communications 2023 14:1* **14**, 1–13 (2023).

47. Tao, R., Sun, Z., Li, F., Fang, W. & Xu, L. Achieving Organic Metal Halide Perovskite into a Conventional Photoelectrode: Outstanding Stability in Aqueous Solution and High-Efficient Photoelectrochemical Water Splitting. *ACS Appl Energy Mater* **2**, 1969–1976 (2019).

48. Wang, M. *et al.* High-Performance and Stable Perovskite-Based Photoanode Encapsulated by Blanket-Cover Method. *ACS Appl Energy Mater* **4**, 7526–7534 (2021).

49. Choi, H. *et al.* Suppression of Undesired Losses in Organometal Halide Perovskite-Based Photoanodes for Efficient Photoelectrochemical Water Splitting. *Adv Energy Mater* **13**, 2300951 (2023).

50. Kim, T. G. *et al.* Monolithic Lead Halide Perovskite Photoelectrochemical Cell with 9.16% Applied Bias Photon-to-Current Efficiency. *ACS Energy Lett* **7**, 320–327 (2022).

51. Chen, Y. W. *et al.* Atomic layer-deposited tunnel oxide stabilizes silicon photoanodes for water oxidation. *Nature Materials 2011 10:7* **10**, 539–544 (2011).

52. Hu, S. *et al.* Amorphous TiO2 coatings stabilize Si, GaAs, and GaP photoanodes for efficient water oxidation. *Science (1979)* **344**, 1005–1009 (2014).

53. Kang, D. *et al.* Printed assemblies of GaAs photoelectrodes with decoupled optical and reactive interfaces for unassisted solar water splitting. *Nature Energy 2017 2:5* **2**, 1–5 (2017).

58. Detz, R. J., Reek, J. N. H. & Van Der Zwaan, B. C. C. The future of solar fuels: when could they become competitive? *Energy Environ Sci* **11**, 1653–1669 (2018).

59. Holmes-Gentle, I., Tembhurne, S., Suter, C. & Haussener, S. Kilowatt-scale solar hydrogen production system using a concentrated integrated photoelectrochemical device. *Nature Energy 2023 8:6* **8**, 586–596 (2023).

60. Jacobsson, T. J., Fjällström, V., Edoff, M. & Edvinsson, T. Sustainable solar hydrogen production: from photoelectrochemical cells to PV-electrolyzers and back again. *Energy Environ Sci* **7**, 2056–2070 (2014).

61. Moss, B., Babacan, O., Kafizas, A. & Hankin, A. A Review of Inorganic Photoelectrode Developments and Reactor Scale-Up Challenges for Solar Hydrogen Production. *Adv Energy Mater* **11**, (2021).

62. Nielander, A. C., Shaner, M. R., Papadantonakis, K. M., Francis, S. A. & Lewis, N. S. A taxonomy for solar fuels generators. *Energy Environ Sci* 8, 16–25 (2014).

64. The European Innovation Council. EIC Pathfinder Challenges 2024 - European Commission. https://eic.ec.europa.eu/eic-funding-opportunities/eic-pathfinder-new-template/eic-pathfinder-challenges-2024_en (2024).

65. Yao, J. *et al.* Quantifying losses in open-circuit voltage in solution-processable solar cells. *Phys Rev Appl* **4**, 014020 (2015).
