# Supplementary material for: Enhanced solar water oxidation and unassisted water splitting using graphite-protected bulk heterojunction organic photoactive layers
Source: Nat Energy. 2025 Mar 18;10(5):581–91. doi: 10.1038/s41560-025-01736-6 (PMC12116381; doi:10.1038/s41560-025-01736-6)
Supplement: Supplementary file 2 — Reporting Summary [file 41560_2025_1736_MOESM2_ESM.pdf]

Corresponding author(s): Dr Salvador Eslava

Solar Cells Reporting Summary

Nature Research wishes to improve the reproducibility of the work that we publish. This form is intended for publication with all accepted papers reporting the characterization of photovoltaic devices and provides structure for consistency and transparency in reporting. Some list items might not apply to an individual manuscript, but all fields must be completed for clarity.

For further information on Nature Research policies, including our data availability policy, see Authors & Referees.

Experimental design

Please check: are the following details reported in the manuscript?

1. Dimensions

Area of the tested solar cells

☒ Yes ☐ No

The information can be found in the figure captions as well as in the "(Photo)electrochemical and solar cell measurements" section in Methods.

Method used to determine the device area

☒ Yes ☐ No

The information can be found in "(Photo)electrochemical and solar cell measurements" section in Methods.
Circular mask with predefined diameters was used:
"...a circular mask with an area of 0.28 cm2."

2. Current-voltage characterization

Current density-voltage (J-V) plots in both forward and backward direction

☐ Yes ☒ No

The hysteresis for organic solar cells is mostly negligible and usually not measured.

Voltage scan conditions
For instance: scan direction, speed, dwell times

☒ Yes ☐ No

The scan rate used for the photoelectrochemical as well as solar measurements is stated in each figure caption separately. The rest of the information can be found in "(Photo)electrochemical and solar cell measurements" section in Methods.

Test environment
For instance: characterization temperature, in air or in glove box

☒ Yes ☐ No

The environment (typically ambient, aqueous 1 M NaOH electrolyte) is stated in each figure caption separately, as well as in the "(Photo)electrochemical and solar cell measurements" section in Methods.

Protocol for preconditioning of the device before its characterization

☐ Yes ☒ No

The devices were not preconditioned before characterization.

Stability of the J-V characteristic
Verified with time evolution of the maximum power point or with the photocurrent at maximum power point; see ref. 7 for details.

☒ Yes ☐ No

Information on the operational stability tests of the photoelectrodes can be found in the figure captions (e.g., Fig. 4) and in "(Photo)electrochemical and solar cell measurements" section in Methods.

3. Hysteresis or any other unusual behaviour

Description of the unusual behaviour observed during the characterization

☐ Yes ☒ No

Not applicable.

Related experimental data

☐ Yes ☒ No

Not applicable.

4. Efficiency

External quantum efficiency (EQE) or incident photons to current efficiency (IPCE)

☒ Yes ☐ No

IPCE spectrum of the highest performing photoelectrode is shown in Fig. 2e. Details of the measurement are provided in the "(Photo)electrochemical and solar cell measurements" section in Methods.

A comparison between the integrated response under the standard reference spectrum and the response measure under the simulator

☒ Yes ☐ No

The integrated photocurrent density under 1 sun illumination is reported in Fig. 2e, while the photocurrent densities of the photoelectrodes under 1 sun illumination are displayed in Fig. 2a and Fig 2b.

For tandem solar cells, the bias illumination and bias voltage used for each subcell

☒ Yes ☐ No

Only monolithic tandem photoelectrodes were measured, meaning that the applied bias and illumination was always the same for the subcells.
The conditions of the measurements are detailed in the figure captions (e.g., Fig. 6).

5. Calibration

Light source and reference cell or sensor used for the characterization

☒ Yes ☐ No

The information can be found in "(Photo)electrochemical and solar cell measurements" section in Methods.

Confirmation that the reference cell was calibrated and certified

☒ Yes ☐ No

The information can be found in "(Photo)electrochemical and solar cell measurements" section in Methods:
"Lot Quantum Design xenon lamp was applied as illumination source with an AM 1.5G filter and a circular mask with an area of 0.28 cm2. 1 sun (100 mW cm-2) irradiance was calibrated by a certified International Light Technologies SEL623 photodetector"

Calculation of spectral mismatch between the reference cell and the devices under test

☐ Yes ☒ No

Minimal spectral mismatch was confirmed by comparing the integrated photocurrent density under 1 sun illumination (reported in Fig. 2e and the photocurrent densities of the photoelectrodes under 1 sun illumination (displayed in Fig. 2a).
Further information can be found in "(Photo)electrochemical and solar cell measurements" section in Methods.

6. Mask/aperture

Size of the mask/aperture used during testing

☒ Yes ☐ No

The information can be found in "(Photo)electrochemical and solar cell measurements" section in Methods.
Circular mask with predefined diameters was used:
"...a circular mask with an area of 0.28 cm2."

Variation of the measured short-circuit current density with the mask/aperture area

☒ Yes ☐ No

The influence of device active area is discussed in the manuscript in the "PEC performance of organic photoanodes" section.

7. Performance certification

Identity of the independent certification laboratory that confirmed the photovoltaic performance

☐ Yes ☒ No

The focus of the work is the photoelectrode performance and so the photovoltaic performance was not certified by an independent certification laboratory.

A copy of any certificate(s)
Provide in Supplementary Information

☐ Yes ☒ No

The focus of the work is the photoelectrode performance and so the photovoltaic performance was not certified by an independent certification laboratory.

8. Statistics

Number of solar cells tested

☒ Yes ☐ No

Supplementary Fig. 13 shows current-voltage curves of 7 organic photoelectrodes from different batches prepared by the same method. Supplementary Fig. 11 shows current-voltage curves of 10 organic solar cells from different batches prepared by the same method.

Statistical analysis of the device performance

☒ Yes ☐ No

Statistical analysis of photoelectrode performance is provided in Fig. 2 b, while statistical analysis of the organic solar cell performance is shown in Supplementary Fig. 11.

9. Long-term stability analysis

Type of analysis, bias conditions and environmental conditions
For instance: illumination type, temperature, atmosphere humidity, encapsulation method, preconditioning temperature

☒ Yes ☐ No

Information on the operational stability tests of the photoelectrodes can be found in the figure captions and in the "(Photo)electrochemical and solar cell measurements" section in Methods.
